# Inducing fluorescence of uranyl acetate as a dual-purpose contrast agent for correlative light-electron microscopy with nanometre precision

**DOI:** 10.1038/s41598-017-10905-x

**Published:** 2017-09-05

**Authors:** Maarten W. Tuijtel, Aat A. Mulder, Clara C. Posthuma, Barbara van der Hoeven, Abraham J. Koster, Montserrat Bárcena, Frank G. A. Faas, Thomas H. Sharp

**Affiliations:** 1 Section Electron Microscopy, Department of Molecular Cell Biology, Leiden University Medical Center, 2300 RC Leiden, The Netherlands; 2 Molecular Virology Laboratory, Department of Medical Microbiology, Leiden University Medical Center, 2300 RC Leiden, The Netherlands; 3 NeCEN, Gorlaeus Laboratories, Leiden University, 2333 CC Leiden, The Netherlands

## Abstract

Correlative light-electron microscopy (CLEM) combines the high spatial resolution of transmission electron microscopy (TEM) with the capability of fluorescence light microscopy (FLM) to locate rare or transient cellular events within a large field of view. CLEM is therefore a powerful technique to study cellular processes. Aligning images derived from both imaging modalities is a prerequisite to correlate the two microscopy data sets, and poor alignment can limit interpretability of the data. Here, we describe how uranyl acetate, a commonly-used contrast agent for TEM, can be induced to fluoresce brightly at cryogenic temperatures (−195 °C) and imaged by cryoFLM using standard filter sets. This dual-purpose contrast agent can be used as a general tool for CLEM, whereby the equivalent staining allows direct correlation between fluorescence and TEM images. We demonstrate the potential of this approach by performing multi-colour CLEM of cells containing equine arteritis virus proteins tagged with either green- or red-fluorescent protein, and achieve high-precision localization of virus-induced intracellular membrane modifications. Using uranyl acetate as a dual-purpose contrast agent, we achieve an image alignment precision of ~30 nm, twice as accurate as when using fiducial beads, which will be essential for combining TEM with the evolving field of super-resolution light microscopy.

## Introduction

Fluorescence light microscopy (FLM) is highly suitable for locating and imaging specific regions of interest within a large field of view. However, FLM yields information only on the fluorescent probe, not on the surrounding cellular context or ultrastructure. Although various super-resolution light microscopy techniques have surpassed the diffraction limit, and routinely achieve resolutions of tens of nanometres^1^, the spatial resolution of conventional FLM is limited to ~200 nm under ideal circumstances^2^, and under cryogenic conditions (cryoFLM), the obtainable resolution is decreased to ~400 nm due to the long working distance lenses required^3^. Transmission electron microscopy (TEM), however, can achieve much higher spatial resolution than FLM, and TEM of stained cellular sections is commonly used for cell biology applications, which allows identification of intracellular membranes and vesicles, as well as large protein complexes such as cytoskeletal elements and nuclear pores^4–6^. However, with the high resolution and wealth of ultrastructural information available when using TEM comes a concomitant decrease in the ease of which regions of interest can be relocated. Combining these advantages of FLM and TEM using correlative light electron microscopy (CLEM) allows the large field of view of FLM to be used to identify rare or transient cellular events for subsequent inspection with the high spatial resolution of TEM^7, 8^.

Correlating data derived from both FLM and TEM modalities relies on accurate alignment of the images. Coarse alignment of identical regions of interest in images derived from both FLM and TEM can be facilitated by using finder grids^8^, or by identifying large morphological features that are visible in both FLM and TEM, such as cell boundaries or nuclei^9, 10^, which can achieve an alignment precision of ~0.5 µm^9, 11^. However, accurately aligning subcellular regions or cytosolic particles to a resolution better than ~0.5 µm is dependent upon identifying discrete particles that are visible in both imaging modalities. Current approaches to accurately align FLM and TEM images include adding fluorescent beads that are also visible by TEM to the cell sections during preparation^6^. However, these beads are stochastically distributed over the surface of the sections and can therefore obscure regions of interest. Furthermore, the accuracy of the resulting alignment is dependent on the presence and distribution of beads; too few or a poor spread of beads can result in a suboptimal image alignment^6, 12^. Alternatively, proteins can be tagged with fluorescent probes that are also visible in the TEM, such as fluoronanogold or quantum dots^13–15^. This approach also relies on an optimal distribution of particles to achieve an accurate alignment, as well as efficient labelling, although not all dual-labelled probes are both fluorescent and electron dense^16^.

The use of green fluorescent protein (GFP), and other endogenously-expressed fluorescent proteins (FPs), results in optimal labelling efficiency, and greatly increased the applicability of FLM to study cellular processes. However, preparing cell sections for TEM requires the use of electron-dense stains, such as heavy-metal compounds, to provide contrast, which are known to quench the fluorescence of fluorophores and therefore limits their use for applications that require CLEM^17, 18^. Typical sample preparation for TEM also includes the use of chemical fixatives and polymers that are autofluorescent, can quench the fluorescence of fluorophores, and induce changes at the ultrastructural level^18–22^. Despite these difficulties, multiple approaches for combining light and electron microscopy have been developed^9, 23^. These include fluorescence imaging prior to fixing and staining the sample for TEM^10, 24^, immunolabelling samples, either before or after (serial) sectioning, which enables fluorescence imaging of fixed and stained material^15, 25^, and the use of probes that remain fluorescent after osmium-staining and fixation^26^. By using dedicated preparation methods utilizing high-pressure freezing, so-called in-resin fluorescence (IRF) protocols, biological samples can be cryo-fixed without the use of chemical fixatives^27^, and the use of a low concentration of uranyl acetate is adequate to provide contrast for TEM and retain sufficient fluorescence of FPs for FLM^6, 28, 29^. The use of IRF methods has allowed the fluorescence of endogenously-expressed FPs to be used for super-resolution light microscopy of cell sections prepared for TEM^30^. Super-resolution CLEM offers numerous advantages over conventional CLEM, such as replacing immunolabelling with endogenously-expressed FPs^30–32^, thereby removing non-specific labelling and eliminating preparation steps. The ability to accurately align FLM and TEM images is therefore critical to realise the potential of super-resolution CLEM. Nevertheless, the lack of visibility of structures or probes visible in both FLM and TEM remains an issue that limits the interpretation of CLEM data to a precision of ~0.5 µm^9, 11^, which is insufficient to unambiguously identify related morphological features at the nanometre resolution attainable.

To develop probes suitable for aligning FLM and TEM images, we turned to the previously reported increase in fluorescence of uranyl-containing compounds at cryogenic temperatures^33, 34^. Reducing the temperature of both solid sediments and solutions of various uranyl-containing compounds has the effect of increasing their fluorescence intensity^35, 36^. We reasoned that this property could be used to induce fluorescence in TEM sections stained with uranyl acetate, a common electron-dense stain used for TEM, by imaging using cryoFLM. CryoFLM usage is becoming more commonplace with the increasing popularity of cryoCLEM^37^, and the development of both home-made^38^ and commercial cryostages^11, 39, 40^. Here, we describe how uranyl acetate stain can be induced to fluoresce using a cryoFLM setup and detected using standard filter sets suitable for imaging GFP, facilitating relocation of the sample in the TEM with nanometre precision. We show that using uranyl acetate as a dual-purpose stain can increase the accuracy and ease of correlation when compared to fluorescent beads, and apply this technique by performing multi-colour CLEM of virus-induced ultrastructural modifications.

## Results and Discussion

### Uranyl acetate can be induced to fluoresce and imaged by cryo-fluorescence light microscopy

**Figure 1:**
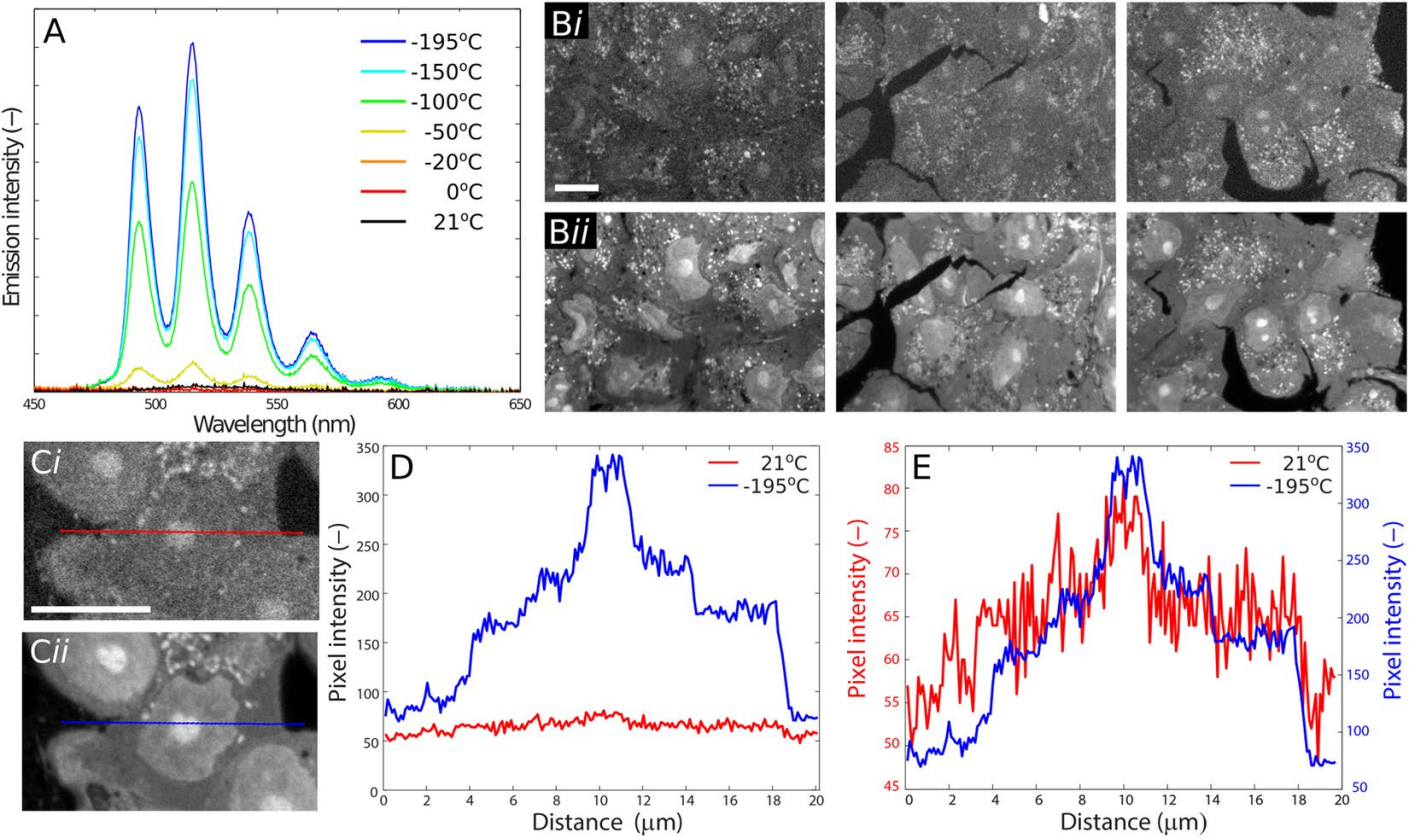
Uranyl acetate can be induced to fluoresce at cryogenic temperatures. (**A**) Emission spectra of uranyl acetate at temperatures between 21 °C and −195 °C. (**B**) Fluorescence microscopy images of Vero E6 cell sections prepared for TEM. The three sections were first imaged at 21 °C (***i***) before being cooled down to −195 °C and imaged again (***ii***), using identical illumination and acquisition parameters. The contrast of each panel is linearly stretched for comparison. (**C**) Comparing the uranyl acetate fluorescence signal from sections of Vero E6 cells imaged at 21 °C (***i***) and −195 °C (***ii***). (**D** and **E**) Line traces of the pixel intensities shown in (**C** ***i***) (red) and (**C** ***ii***) (blue). Absolute pixel values are shown in (**D**), and pixel values plotted on separate axes in (**E**). All scale bars represent 10 µm.

To assess the increase in fluorescence intensity of uranyl acetate at cryogenic temperatures, we used a custom-built spectrometer setup to measure the spectrum of uranyl acetate dissolved in water (1% w/v) at various temperatures (Fig. 1A). After excitation at 405 nm, we collected emission spectra from 350 nm to 900 nm. We found that the fluorescence of uranyl acetate could be detected at 21 °C, although at very low intensity (black line, Fig. 1A). Below −50 °C the signal from uranyl acetate became more intense (yellow line, Fig. 1A), and below −100 °C five distinct vibrational states within the spectra could be clearly resolved, with a maximum emission peak at 515 nm, and smaller peaks at 493, 538, 564 and 591 nm (cyan and blue lines, Fig. 1A), in broad agreement to values reported for other uranyl-containing compounds^35^.

Next, we imaged samples prepared for TEM and stained with uranyl acetate, a common electron-dense contrast agent, by cryoFLM. Vero E6 cells were fixed by high-pressure freezing and prepared using the aforementioned in-resin fluorescence (IRF) protocol, which includes 0.1% uranyl acetate in the freeze-substitution medium. We imaged the sections at both 21 °C and −195 °C using a Linkam cryostage with a long working-distance lens that is compatible with cryoFLM imaging^3^. Based on the emission spectrum of uranyl acetate, fluorescence could be imaged using common filter sets for GFP. At 21 °C, a 200 nm-thick section of a population of Vero E6 cells exhibited minimal fluorescence (Fig. 1B*i*), which revealed the outline of cells and nucleoli, as well as puncta within the cellular cytoplasm. In contrast, after cooling the cryostage to −195 °C with liquid nitrogen, refocusing on the same area and acquiring images using identical illumination and acquisition parameters, the cells were brightly fluorescent (Fig. 1B*ii*). The increased fluorescence intensity at −195 °C revealed the location of cellular and nuclear membranes, as well as more defined nucleoli and organelles. This increased signal-to-noise ratio is visible in the line plot shown in Fig. 1C–E, which clearly resolves the boundaries of the cell, nucleus and nucleolus in raw images at −195 °C. We performed all subsequent FLM imaging at cryogenic temperatures to exploit this superior signal-to-noise ratio and brightness, except where otherwise indicated.

To determine whether this fluorescence is due solely to uranyl acetate and not to any other phenomenon, we imaged cell sections prepared using an identical protocol but without the addition of uranyl acetate during freeze substitution. Although there was detectable fluorescence in sections prepared without any heavy-metal stain (Supplementary Fig. S1), this fluorescence was not localised to any detectable structure and is likely due to autofluorescence of the cells^41^.

To our knowledge, the use of uranyl acetate as a fluorescent probe for cell biology has not been previously reported. Typically, sections prepared for TEM using the IRF protocol are imaged using an objective lens with a high numerical aperture (NA), which requires immersion oil and the sample must therefore be sandwiched between two glass coverslips to avoid oil-contamination of the TEM grid^6^. To prevent the sections sticking to the glass, grids are immersed in aqueous media between the coverslips. When we performed this approach, we did not detect any fluorescence from cell sections other than a uniform weak autofluorescence visible in all colour channels (data not shown). This may be because the aqueous environment provides a non-radiative decay mechanism that inhibits the exited uranyl acetate chromophore from fluorescing^42^, which we avoid by imaging our grids dry using the Linkam cryostage with an air-objective lens.

### Using uranyl acetate as a dual-purpose stain for cryoFLM and TEM

**Figure 2:**
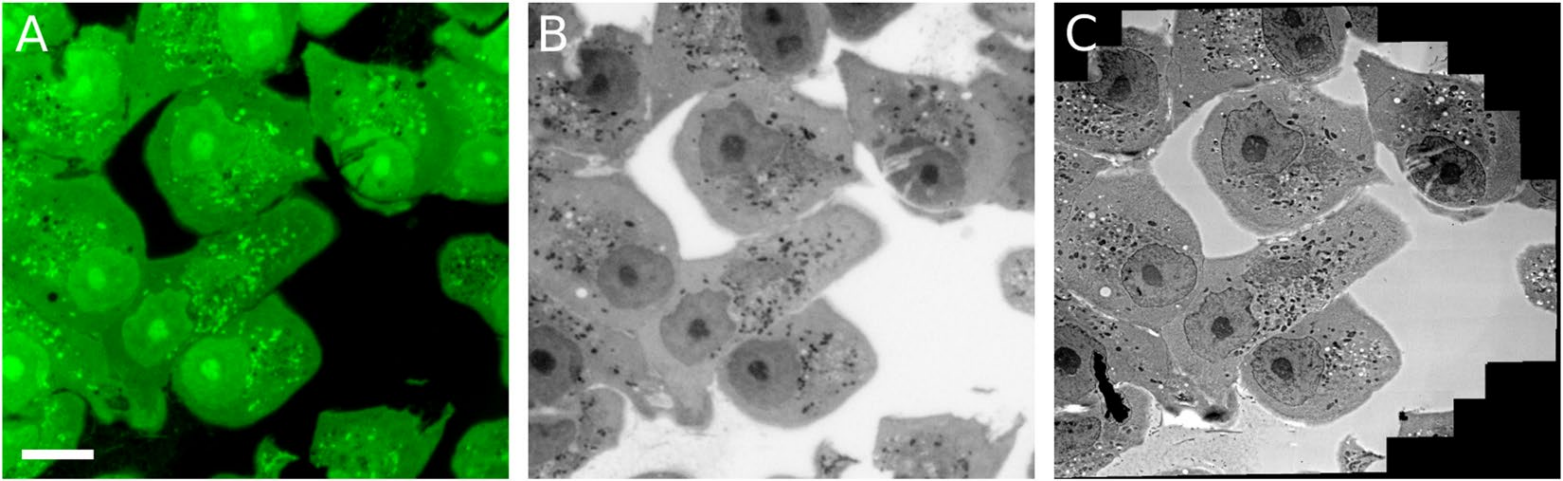
Uranyl acetate as a dual-purpose fluorescent and electron dense stain for correlative light electron microscopy. (**A**) Fluorescence microscopy image at −195 °C of Vero E6 cell sections prepared for IRF. (**B**) Inverted grey-scale fluorescence microscopy image of (**A**). (**C**) TEM image of the same region as panels A and B. Scale bar represents 10 µm and applies to all panels.

Aligning images derived from FLM and TEM requires features or probes that can be detected with both imaging modalities: uranyl acetate shows promise as it provides both a fluorescent and electron dense stain. Figure 2 shows a 200 nm-thick section of a population of Vero E6 cells prepared using the IRF method. At −195 °C the cells, cytosol, organelles and nuclei were brightly fluorescent (Fig. 2A). After thawing the section, imaging the cell section by room temperature TEM reveals the ultrastructural detail stained by uranyl acetate (Fig. 2C), which is the only electron-dense contrast agent present. Converting Fig. 2A to grey-scale and inverting the contrast (Fig. 2B) to match that of the TEM image (Fig. 2C) reveals a similar stain distribution: i.e., regions that are fluorescent are also electron dense, further supporting the conclusion that the fluorescence is due to uranyl acetate. We observed no change in sample quality when we performed TEM on sections before and after cooling and thawing (data not shown), indicating that imaging by cryoFLM prior to TEM does not adversely affect the stained cellular ultrastructure.

Using uranyl acetate as a dual-purpose contrast agent enables the use of FLM to reveal the cellular context, not just fluorescently-labelled subcellular regions. This makes navigation and interpretation of TEM samples by FLM faster and more intuitive, and can therefore greatly assist with identifying regions of interest for relocation in the TEM.

### Uranyl acetate can be used to align cryoFLM and TEM images with nanometre accuracy

**Figure 3:**
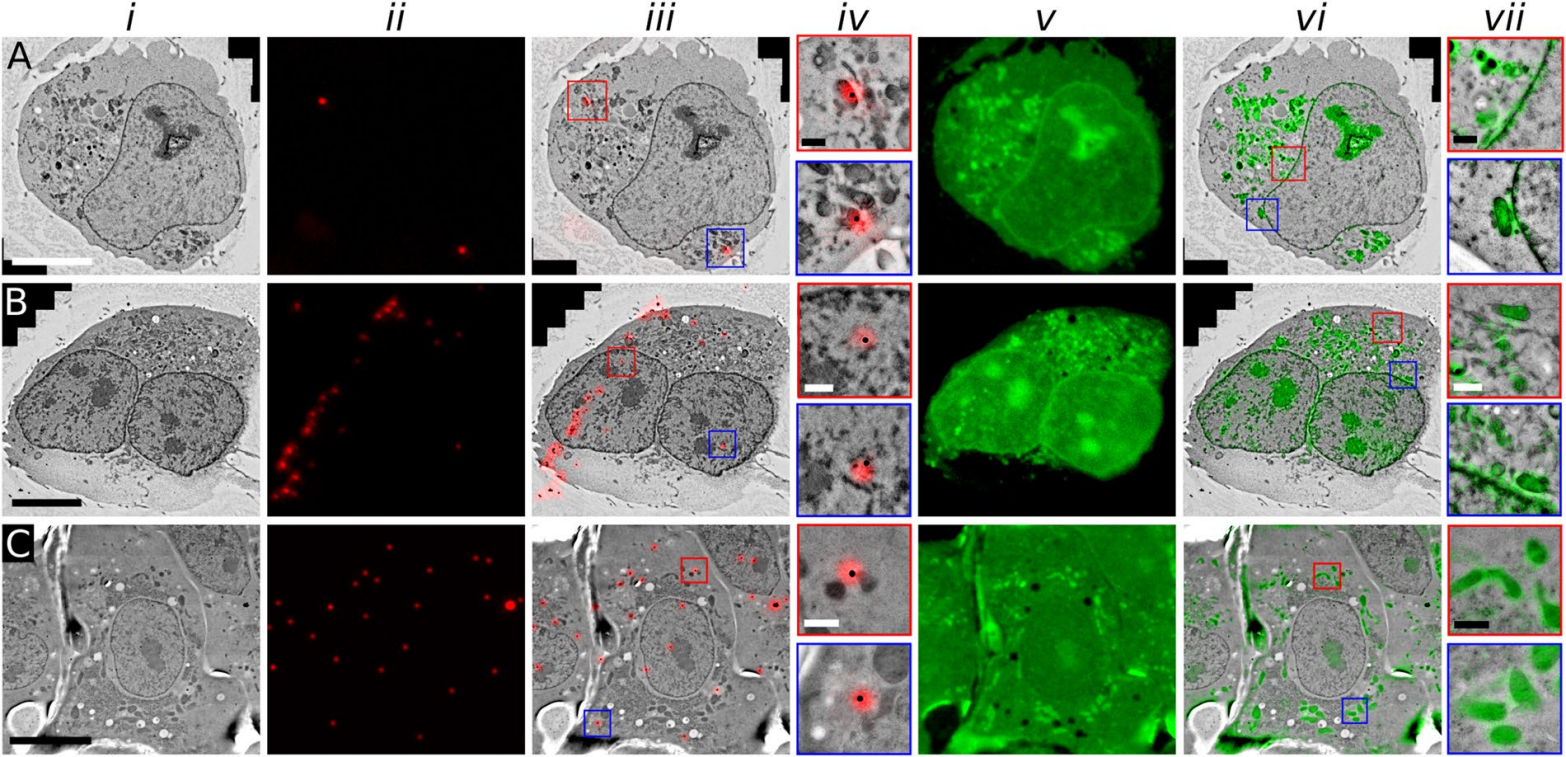
Aligning cryoFLM and TEM images using either fiducial beads or uranyl acetate. TEM and cryoFLM images of Vero E6 cells, prepared with the IRF sample protocol and labelled with 200 nm red fiducial beads. (**A**) Section with sparse distribution of fiducial beads, (**B**) where the fiducial beads are mostly oriented along a single orientation and (**C**) with a widely-spread distribution of fiducials. Panel (***i***) shows the TEM image, (***ii***) cryoFLM image of fiducial beads (red), (***iii***) an overlay of ***i*** and ***ii***, and panel (***iv***) shows magnified areas indicated by the coloured boxes in ***iii***. Panels (***v***–***vii***) show the cryoFLM image of uranyl acetate (***v***), its overlay with the TEM image (***vi***) and magnified areas indicated by the coloured boxes (***vii***) indicated in (***vi***). The image in (**A**) was cropped for visualisation and clarity, for full image see Supplementary Fig. S2. Scale bars for (***i***–***iii***) and (***v***–***vi***): 10 µm; scale bars for (***iv***) and (***vii***): 1 µm.

FLM and TEM images can be aligned with an accuracy of ~50–100 nm with the use of beads that are visible in multiple fluorescent channels as well as in TEM images^6, 12^. As discussed in the *Introduction*, the use of fiducial markers is not ideal; an extra processing step is required to add the beads to the sample, and fiducial beads can obscure regions of interest in the sample. Furthermore, the spread of the fiducial beads is inherently stochastic, but a good distribution of beads around the region of interest is required for accurate alignment. This is illustrated in Fig. 3, which shows 200 nm thick sections of Vero E6 cells that were incubated with fluorescent beads after processing using the IRF method. The beads are stochastically dispersed, and hence in some regions the beads are not well distributed (Fig. 3A), whilst in other regions the beads are closely packed in one predominant direction (Fig. 3B). Each TEM image (*i*) in Fig. 3 was used to align either the signal from the fiducial beads (*ii*) or the uranyl acetate (*v*) of the corresponding cryoFLM image using either fiducial-based alignment (*iii*) or whole-image registration using the uranyl acetate stain (*vi*). For fiducial-based alignments of FLM and TEM images, we manually selected up to 20 beads (if present) that were visible in both modalities and aligned the images using eC-CLEM^43^. This fiducial-based alignment approach relies on unambiguous determination of which bead should be selected and paired in each modality. However, the higher resolution of the TEM image can reveal more than one bead within the lower resolution point-spread function of the FLM image (see e.g., Supplementary Fig. S4), leading to misidentified beads. Utilizing a leave-one-out cross validation (LOOCV) scheme to measure the difference between the coordinates of the predicted and the true position of each fiducial^44^, as described by Kukulski *et al*.^6^, gave us a measure of correlation accuracy for each bead distribution scheme (Table 1 and Supplementary Table S1). Figure 3A shows a situation with sparse numbers of fiducials (see also Supplementary Fig. S2); with only 4 fiducials present, the accuracy of alignment was 333 nm (Supplementary Table S1 and Supplementary Fig. S3). Instances where there are an adequate number of beads but they are spaced along a predominant direction tend to yield an anisotropic alignment accuracy: Fig. 3B shows such an instance, and whilst the overall accuracy was 112 nm, the outlier identified in Fig. 3B*iv* (blue boxed region), is only within 352 nm of the predicted position (see also Supplementary Fig. S4 and Supplementary Table S1). Finally, Fig. 3C shows a situation with well-distributed beads, which gave an accuracy measure of 64 nm (see also Supplementary Fig. S5 and Supplementary Table S1).

**Table 1:** An overview on the accuracy of alignment for the images shown in Figs 3–6.

Figure	3 A	3B	3 C	4	5	6
Number of beads picked for LOOCV	4*	20	20	—	—	—
Accuracy of alignment (LOOCV)	333 nm	112 nm	64 nm	—	—	—
Number of structures analyzed for uranyl acetate based alignment	10	10	10	10	10	10
Accuracy of alignment, based on uranyl acetate	26 nm	30 nm	25 nm	21 nm	38 nm	36 nm
Alignment of FP to uranyl acetate (FP variant in brackets)	—	—	—	12 nm (GFP)	24 nm (RFP)	50 nm (RFP)
Overall alignment accuracy	26 nm	30 nm	25 nm	24 nm	45 nm	62 nm

*Only two beads are shown in the field of view, for full image, please refer to Supplementary Fig. S2. For further details on the alignment accuracy, please refer to Supplementary Tables S1–S4, as well as Supplementary Figs S2–S8 & S10–S15.

Utilising uranyl acetate as a dual-purpose stain allowed us to align the FLM and TEM images without the need to manually pick beads or rely on stochastic fiducial distribution. We used the software program Elastix^45^ to perform image registration based on normalised mutual information. After registration, images were transformed using an affine transformation to align cryoFLM images of uranyl acetate-stained sections (Fig. 3*v*) to the TEM images (Fig. 3*vi*). The presence of uranyl acetate throughout the sample can be exploited by Elastix to allow for local corrections to adjust for electron-beam induced damage to the sample, or indeed sample shrinkage. Whilst this use of non-rigid warping may result in higher-accuracy registration, as well as further possible local improvements at higher magnifications, we did not find this necessary for accurate registration. Because the crowded cellular environment is visible in the uranyl acetate image, it was not possible to identify isolated fiducial beads suitable for assessing the alignment of the uranyl acetate and TEM images using LOOCV. Instead, we determined the accuracy of alignment by treating identifiable cellular structures as fiducial markers (Supplementary Fig. S6-S8). Plotting a line through each structure allowed us to determine the full-width-half maximum (FWHM) of the intensity visible in both cryoFLM and TEM images. The average offset between each FWHM yielded alignment accuracies between 25 and 38 nm, with an average of 30 nm (Table 1 and Supplementary Tables S2 and S4).

Our data demonstrates that whole-image registration utilizing uranyl acetate fluorescence yields image alignments that are more than twice as accurate as when an optimal distribution of fiducials is available, 31 nm (avg.) *cf*. 64 nm, respectively (Table 1). Moreover, the uranyl acetate is always present throughout the region of interest, so that accurate alignment can be consistently performed. Our uranyl acetate-based alignment approach has several advantages over other correlation methods: fiducial markers such as fluorescent beads are not required; image deformations, such as those arising from a tilted specimen or large stitched images, can be corrected for with high fidelity; and correlations can be achieved in areas without fluorescent proteins or markers (see below). This increased correlation accuracy will be essential for accurately aligning super-resolution images collected using single-molecule localization methods, such as photoactivated localization microscopy (PALM)^46^ or stochastic optical reconstruction microscopy (STORM)^47^, with TEM images^48–52^.

### Uranyl acetate fluorescence at cryogenic temperatures can be used for multicolour CLEM

**Figure 4:**
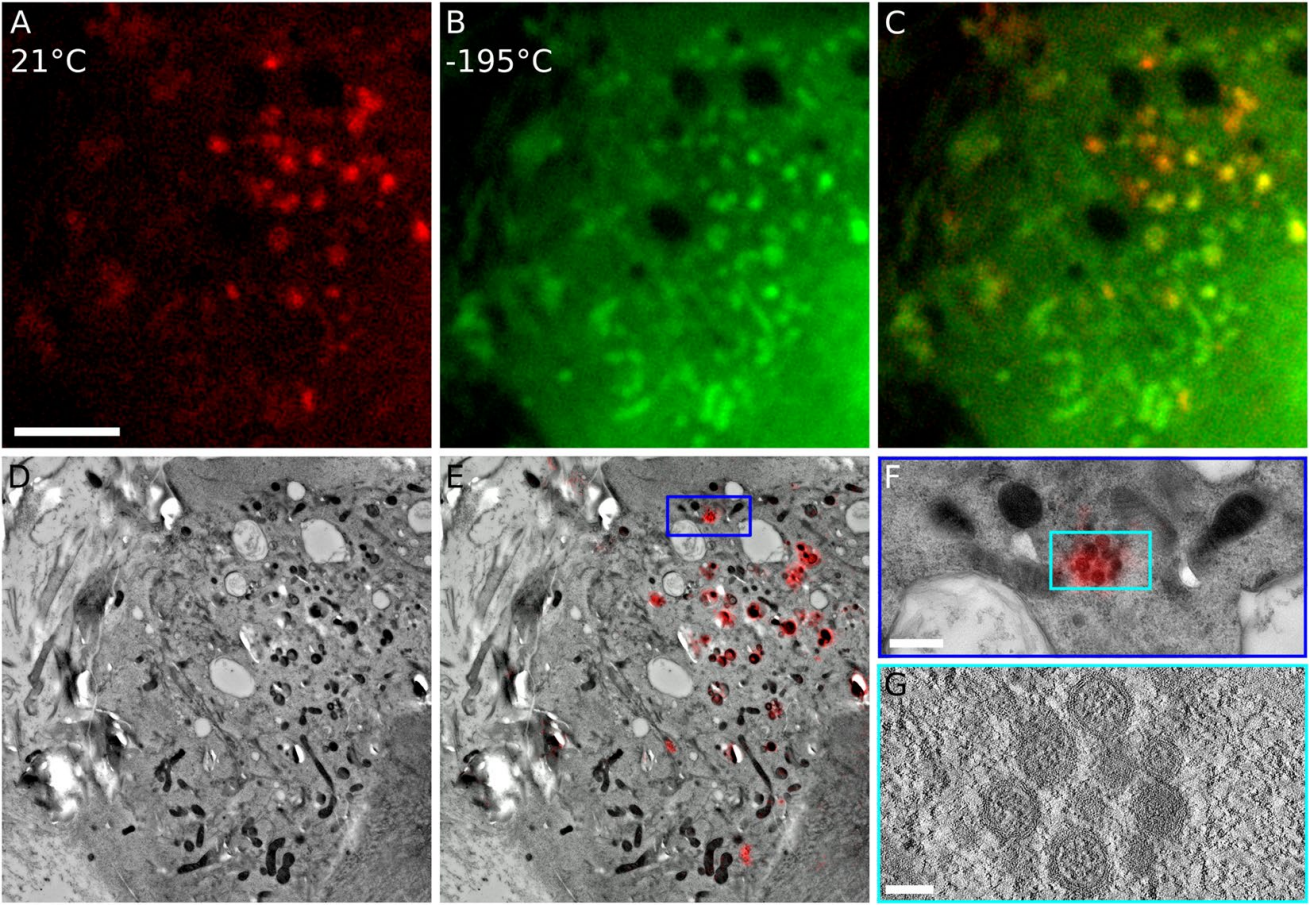
Fluorescence from both GFP and uranyl acetate can be imaged in the same section. (**A**) The fluorescent signal from GFP (red) can be acquired at 21 °C in sections of E6 Vero cells expressing nsp3-GFP. (**B**) Uranyl acetate fluorescence acquired at −195 °C. (**C**) Merging the signals from GFP (red) and uranyl acetate (green) allows GFP puncta to be visualized within the cellular ultrastructure. (**D**) TEM image of the cell sections shown in (**A**–**C**). (**E**) GFP signal (red) aligned to the TEM image with a precision of 12 nm using the signal from uranyl acetate. (**F**) High magnification image of the blue boxed region in (**E**). (**G**) A 10 nm-thick slice through a tomographic volume acquired at the cyan-boxed region in (**E**) showing a cluster of double-membrane vesicles. Scale bars: 5 µm in (**A**–**E**), 500 nm in (**F**), and 100 nm in (**G**).

Using standard GFP filter sets to image uranyl acetate enables multicolour fluorescence imaging. Because GFP, and variants thereof, are perhaps the most widely-used endogenous fluorescent tags used in cell biology, we sought to develop our method to enable imaging of uranyl acetate and GFP-tagged proteins within the same section. Although the emission spectrum of uranyl acetate (green, Supplementary Fig. S9) overlaps with that of GFP (cyan, Supplementary Fig. S9), our data suggests that it should be possible to distinguish between these fluorophores in sections prepared for TEM; whilst GFP can be imaged at 21°C in cell sections prepared for TEM^6^, uranyl acetate is not brightly fluorescent above −100 °C (Fig. 1). To exploit this, we prepared 200 nm-thick cell sections for IRF that contained Vero E6 cells expressing an equine arteritis virus (EAV) protein, non-structural protein-3 (nsp3), fused to GFP. EAV is known to induce membrane modifications in infected cells in the form of double-membrane vesicles, which are involved in virus replication^53, 54^. Imaging the sections at 21°C revealed fluorescent puncta (red, Fig. 4A) corresponding to the subcellular location of nsp3-GFP, and in agreement with previous observations^53, 54^. When the same section was cooled to −195 °C and imaged with the same filter sets, ultrastructural fluorescence associated with uranyl acetate was clearly visible (green, Fig. 4B). There was sufficient information in both the GFP and uranyl acetate images to use Elastix to directly align the FLM images (Fig. 4C) with an accuracy of 12 nm (Table 1 and Supplementary Table S3) . The resulting merged image shown in Fig. 4C displays the location of GFP-tagged proteins within the cellular ultrastructure stained by uranyl acetate. Using the signal from uranyl acetate allowed us to align GFP fluorescence to TEM micrographs (Fig. 4D) with an overall accuracy of 24 nm (Table 1 and Supplementary Table S3 & S4). The resulting image revealed that GFP fluorescence (red, Fig. 4E) precisely co-localised with viral-associated membrane modifications (Fig. 4E,F). Inspection of a 10 nm-thick slice through an electron tomogram clearly showed double-membrane vesicles characteristic of EAV infection^53^ (Fig. 4F). Thus, serial imaging of cell sections at 21°C prior to cooling to −195 °C enables localisation of GFP-tagged proteins in TEM micrographs with 24 nm accuracy by using uranyl acetate fluorescence to precisely align FLM and TEM images.

**Figure 5:**
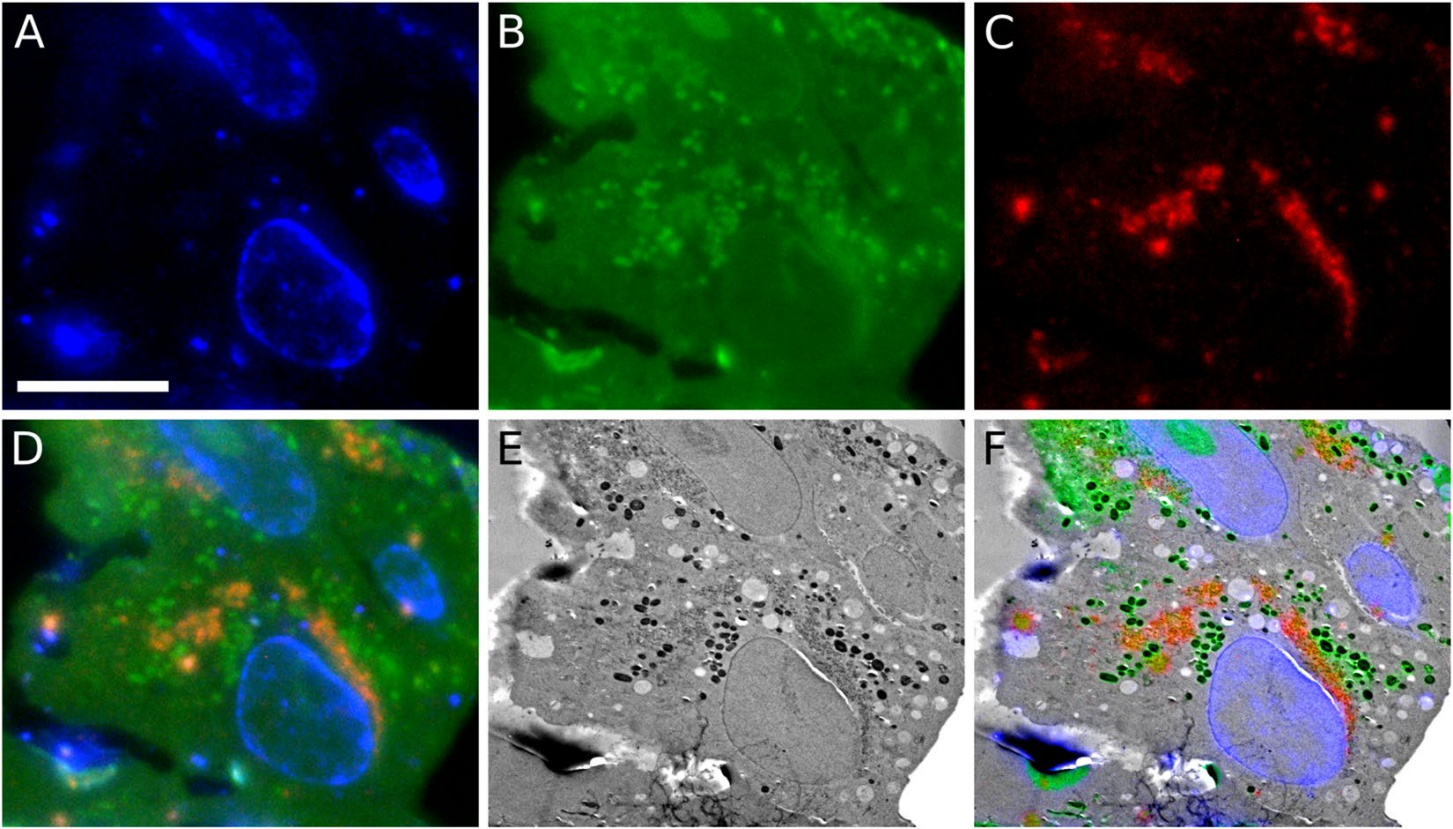
Uranyl acetate staining is compatible with multicolour cryoFLM. (**A**–**D**) Three colour images of sections of Vero E6 cells infected with EAV-RFP2, showing DAPI stain (blue, **A**), uranyl acetate (green, **B**), and RFP (red, **C**), and the merged channels (**D**). (**E**) TEM image corresponding to the cells shown in (**A**–**D**). (**F**) Aligned FLM and TEM images. Scale bar: 10 µm and applies to (**A**–**F**).

To demonstrate the compatibility of uranyl acetate fluorescence with other fluorophores for multicolour imaging, we imaged Vero E6 cells that had been infected with a recombinant EAV virus (EAV-RFP2) that, upon infection, expresses the viral non-structural protein-2 (nsp2) genetically fused to red fluorescent protein (RFP). After infection and processing for IRF to retain the RFP fluorescence, samples were post-stained with DAPI and imaged at −195 °C. The fluorescent channels of DAPI, uranyl acetate and RFP are distinct (Fig. 5A,B and C, respectively, and Supplementary Fig. S9) and, when aligned and merged, clearly show the location of the nucleus (blue), viral nsp2 protein (red) and underlying cellular ultrastructure (green) (Fig. 5D). Using the signal from uranyl acetate allowed us to align the FLM and TEM images (Fig. 5F) with an accuracy of 38 nm (Table 1 and Supplementary Table S4). Aligning the different FLM colour channels to the uranyl acetate signal is a necessary step for high-accuracy alignment to TEM images, especially when using cryoFLM, which is known to be more susceptible to drift^12^. To demonstrate image alignment without requiring fiducial beads, we aligned both the DAPI and RFP channel to the uranyl acetate channel independently based on structures visible in both channels (Supplementary Figs S12 and S13). These structures were used to determine a rigid translation to align the images, which is sufficient to achieve high accuracy alignment of FLM images^12^. By plotting the respective translation required to align each structure to the uranyl acetate image, we were able to ensure identical, point-like structures visible in the uranyl acetate (green) channel were selected for the DAPI (blue) and RFP (red) channels (Supplementary Figs S12 and S13). We determined the alignment accuracy for the different colour channels by calculating the standard deviation of the spread of the channel offsets, which were measured as 24 nm and 106 nm for the RFP and DAPI channel, respectively (Supplementary Fig. S13). Combing these alignment accuracy values with those of uranyl acetate, as described by Schellenberger *et al*.^12^, resulted in an overall alignment accuracy of RFP to the TEM image of 45 nm (Table 1).

**Figure 6:**
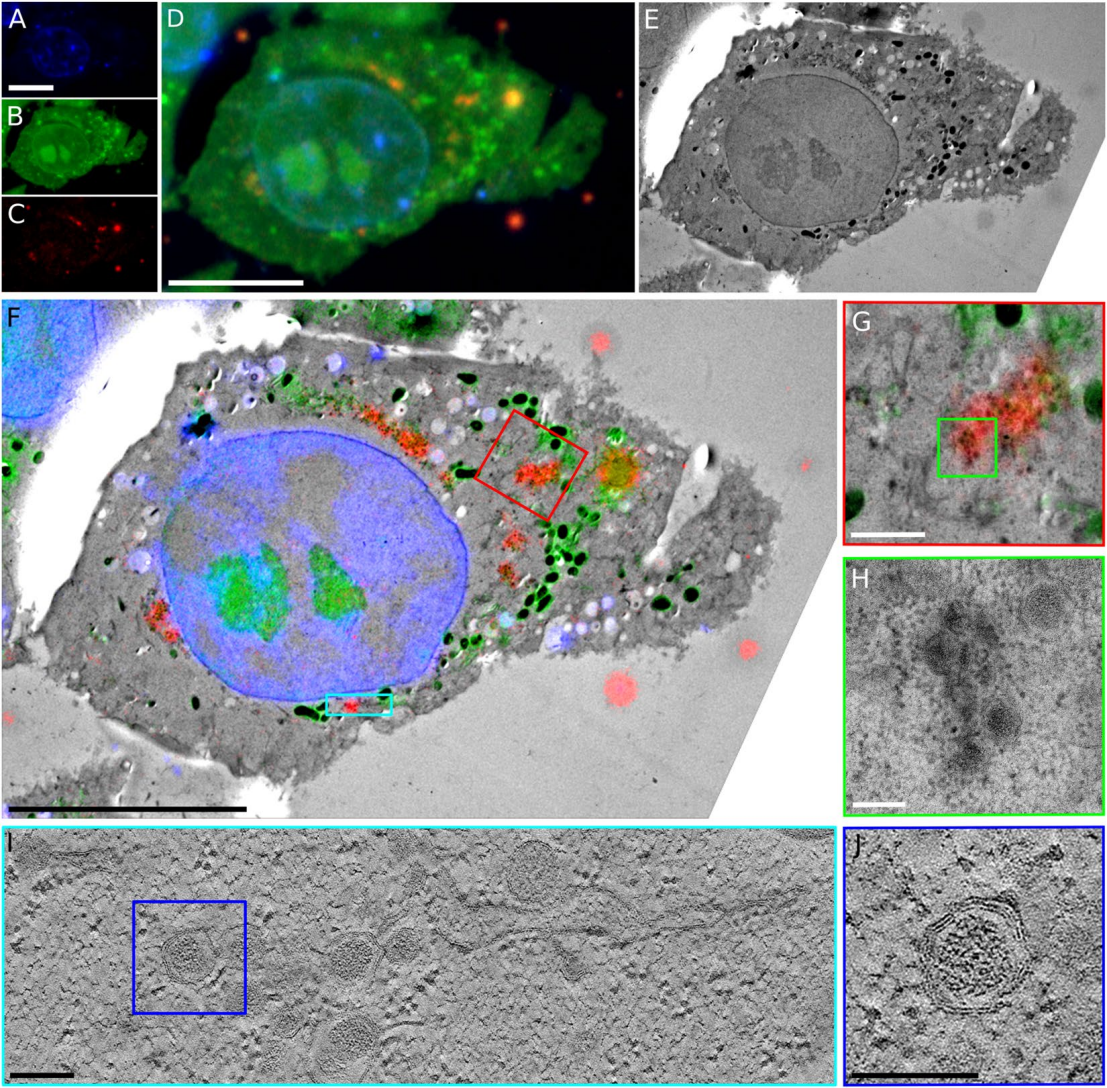
Three-colour CLEM using uranyl acetate to localise virus-induced membrane rearrangements. (**A**–**D**) Three colour images of sections of E6 Vero cells infected with EAV-RFP2, showing DAPI stain (blue, **A**), uranyl acetate (green, **B**), RFP (red, **C**), and the merged channels (**D**). (**E**) TEM image of the same cell shown in (**A**–**D**). (**F**) Overlay of the three-colour cryoFLM images with the TEM image. (**G**) A higher magnification image of the red-boxed region in (**F**). (**H**) A higher magnification image of green-boxed region in (**G**), showing the typical morphology of a region with virus-induced double-membrane vesicles. (**I**) A 10 nm-thick slice through a tomographic volume acquired at the cyan-boxed region in (**F**). (**J**) Higher magnification view of the blue-boxed region in (**I**) showing the typical morphology of a double-membrane vesicle. Scale bars: 10 µm in (**A**–**F**), 1 µm in (**G**) and 150 nm in (**H**,**I** and **J**).

Using the signal of uranyl acetate at cryogenic temperatures to correlate multi-colour fluorescence images with TEM micrographs allowed us to perform high-precision CLEM of EAV-infected Vero E6 cells. Figure 6 shows infected cells imaged by cryoFLM (Fig. 6A–D) that have been relocated in the TEM (Fig. 6E). The TEM image was aligned to the signal from uranyl acetate with an accuracy of 36 nm (Table 1 and Supplementary Table S4), which allowed us to align the RFP signal to the TEM image with an accuracy of 62 nm without the use of fiducial markers (Table 1 and Supplementary Figs S14 and S15). After alignment, the images allowed virus-induced morphological changes to be quickly detected and investigated at high resolution (Fig. 6F–H), which clearly show regions of virus-induced membrane modifications (Fig. 6H) at the location of the red fluorescent signal (Fig. 6G), in agreement with previous observations^53^. We acquired a tomogram at the cyan area identified in Fig. 6F and reconstructed a 200 nm-thick volume of the cell section. A 10 nm-thick central slice from the tomogram clearly shows the double-membrane vesicles containing an electron dense core, typical of EAV-infected cells^53^, that coincide with the RFP-tagged viral protein nsp2 (Fig. 6I and J).

We have shown that using both GFP and RFP can enable multicolour imaging using endogenously-expressed FPs (Figs 4, 5 and 6), and that imaging at −195 °C reduces the bleaching rate of RFP (Supplementary Fig. S16), which enables higher intensity imaging and an improved signal-to-noise ratio, as described previously for GFP^55^, YFP^56^ and other fluorophores^51, 57, 58^. Multicolour imaging may also be extended by using probes that fluoresce in the far red ( >650 nm; Supplementary Fig. S9), such as AlexaFluor 680 or the FP mGarnet^59^, or elaborated by performing on-section labelling with fluorophores^50^.

## Conclusions

By exploiting the increased brightness of uranyl acetate at cryogenic temperatures, we have established that uranyl acetate can be used as a dual-purpose contrast agent for both TEM and cryoFLM. The equivalent staining in each imaging modality allows cell sections prepared for TEM to be imaged by FLM, which makes navigation and interpretation of the samples faster and more intuitive, and enables FLM and TEM images to be aligned with nanometre precision. Furthermore, this accuracy can be obtained without the need to add fiducial beads, or other exogenous markers, to the sample.

Using uranyl acetate as a dual-purpose contrast agent is compatible with the use of GFP- and RFP-tagged intracellular proteins. We have shown that GFP fluorescence can be distinguished from that of uranyl acetate by imaging cell sections first at 21°C before performing cryoFLM, which enabled identification of viral protein-induced intracellular membrane modifications in TEM images with high precision. Furthermore, we performed three-colour CLEM to localize the subcellular location of the arteriviral protein nsp2 in EAV-RFP2-infected Vero E6 cell sections. TEM images and 3-dimensional tomograms were acquired after image correlation at the regions indicated by RFP fluorescence, revealing virus-induced double-membrane vesicles.

The use of uranyl acetate as a general contrast agent for CLEM can be incorporated into commonly-used sample preparation protocols for TEM, and as such we envisage this approach will be utilised by numerous laboratories requiring accurate correlation of light and electron microscope images, which will be essential for combining TEM with the rapidly-developing field of super-resolution microscopy.

## Materials and Methods

### Spectroscopy of uranyl acetate

Uranyl acetate (Sigma Aldrich) was dissolved in water at 1% (w/v). We adapted a Linkam cryostage (THMS 600, Linkam Scientific, UK) to serve as a spectrometer capable of measuring the spectra of samples at variable temperatures. Excitation of the sample with a 405 nm laser (LuxX + 405–120, Omicron-Laserage Laserprodukte GmbH, Germany) was achieved via a side window of the lid of the cryostage. Light was collected close to the sample using an aspheric lens (A397TM-A, Thorlabs Inc, USA) coupled into a multimode optical fibre (M15L01, Thorlabs Inc, USA), and measured with an AvaSpec-2048L Fibre-optic spectrometer (Avantes, the Netherlands). The uranyl acetate solution was loaded into a quartz cuvette (Linkam Scientific, UK), and cooled to −195 °C within the cryostage. The spectrum was measured again before the temperature was increased to 21 °C (room temperature), during which the spectrum was measured at intermediate temperatures. The spectra of DAPI, RFP, GFP and Alexa Fluor 680 were obtained from the online SpectraViewer from Thermoscientific (https://www.thermofisher.com/order/spectra-viewer).

### Cell culture and infection

For GFP expressing cells, EAV polyprotein constructs were assembled in pDONR201 based on the previously described full-length cDNA clone of EAV pEAN511^60^. Constructs for the expression of nsp2 and nsp3 (pcDNA3) contained amino acids 261–1063 of EAV pp1a with an HA-tag fused to the N-terminus of nsp2. The C-terminus of nsp3 lacked the final glutamic acid of nsp3 and was instead fused to eGFP. Vero E6 cells were transfected using AMAXA electroporation with 2 µg of pcDNA3 for each 1 × 10^6^ cells. For infection with EAV-RFP2, Vero E6 cells were cultured on sapphire disks as described^53^, before being infected with a recombinant EAV (EAV-RFP2), which expresses the red fluorescent protein (RFP) variant TagRFP attached to nsp2 and was built following the same design as the previously described EAV-GFP2^61^. Infections with wild-type and recombinant EAV were carried out at 39.5°C with a multiplicity of infection of 10.

### High-pressure freezing and freeze-substitution

Samples were prepared for microscopy using a protocol modified from Kukulski *et al*.^6^, which preserves sufficient fluorescence of FPs for FLM^6, 62^. Samples of transfected or infected cells grown on sapphire disks (Wohlwend, Germany) were high-pressure frozen using a Leica EM PACT-2 (Leica Microsystems, Germany). Infected cells were fixed after 7 hours post-infection. Freeze substitution was performed in a Leica EM AFS2 during which samples were infiltrated with fixative (acetone with 0.1% (w/v) uranyl acetate) for 46 hours at −90°C before the temperature was raised to −45°C over the course of 9 hours. Samples were maintained at −45°C for 5 hours before being washed three times with acetone for ten minutes. Infiltration with Lowicryl HM20 (Polyscience Inc.,USA) was achieved over 16 hours; initial infiltration with 15% Lowicryl (v/v) was performed over 4 hours, before the concentration was increased to 25%, 50% and finally 75% in 4 hour increments. During the final two steps, the temperature was raised to −35°C and −25°C, respectively. Next, infiltration with 100% Lowicryl was performed three times for 10 minutes each, with agitation, also at −25°C. Finally, Lowicryl polymerisation of the HM20 was achieved using UV illumination for 48 hours before the temperature was raised to 20°C over the course of 4 hours and held at 20°C for a further 24 hours, also with constant UV illumination. Sample blocks were sectioned to a thickness of 200 nm using an Ultracut S ultramicrotome (Leica Microsystems), and sections were picked up on continuous carbon finder grids (Electron Microscopy Science, USA).

### Fluorescence microscopy

To stain cell nuclei, cell sections were incubated for 15 minutes with nuclear stain 4′ 6-diamidino-2-phenylindole dihydrochloride (DAPI; Sigma Aldrich), and washed 3 times with deionized water. Where indicated, 200 nm fluorescent beads (FluoSpheres #F8807, Molecular probes) were added to the samples by incubating grids on a 1:20,000 dilution in deionized water for 5 minutes, and washed 3 times with deionized water. Fluorescence imaging was performed on a Zeiss Axio Imager M2 (Zeiss, Sliedrecht, the Netherlands), using a 100× magnification achromatic objective lens with a numerical aperture of 0.75 and a working distance of 4 mm (LD EC Epiplan-Neofluar, Zeiss). The microscope was equipped with a Linkam cryostage (CMS-196, Linkam Scientific Ltd, Chilworth). Grids were first mounted in the cryostage before being cooled down to -195°C using liquid nitrogen. The fluorescence of uranyl acetate and GFP were detected using a standard filter set (filter set 38, Endow GFP shift free (E), Zeiss). Filter sets 49 and 20 (Zeiss) were used for the detection of DAPI and RFP, respectively. Exposure times of 3 seconds were used for the detection of uranyl acetate and GFP, whilst exposure times of 3–10 seconds were used for detection of RFP. After imaging, samples were taken from the cryostage, thawed, and stored at room temperature prior to imaging by electron microscopy.

### Transmission electron microscopy and tomography

For TEM, grids were loaded in a room temperature single-tilt holder (FEI Company, USA), and inserted into a Tecnai T12 transmission electron microscope (FEI Company, USA) operating at 120 kV, equipped with either an Eagle CCD camera (FEI Company, Eindhoven, NL) or a OneView CMOS detector (Gatan Inc., Pleasanton, USA). Large tile-scans were acquired as described by Faas *et al*.^63^ using a defocus of −1 μm and a nominal magnification of 6,500× . Images were binned 2× , corresponding to a final pixel size of 3.3 nm. Tomographic tilt series were acquired using the software package Xplore3D (FEI Company, Eindhoven, NL), at a nominal magnification of 21,000× , and were binned 2× for a final pixel size of 1 nm. Automatic focussing to −2 µm was performed prior to each image acquisition. A linear tilt scheme was used from −60° to + 60° in 2° increments. Tomograms were reconstructed with the software package IMOD^64^ using patch-tracking alignment and weighted back-projection.

### Alignment and visualisation of CLEM overlays

The software program Elastix^45^ was used to align cryoFLM images of uranyl acetate-stained sections to TEM images, as well as to align TEM images of different magnifications. Registration was based on maximising the normalised mutual information^45^, and the registered images were transformed using an affine transformation. Masks were automatically generated to exclude areas with pixel values of zero, which were then not used for image registration. Different colour channels in the FLM were aligned by rigid translation based on aligning between 4–11 points picked that were visible in the different colour channels. For multi-colour CLEM, the resulting transformation from Elastix was applied to the different colour channels using the “transformix” feature within Elastix. For final visualisation, aligned images were loaded into Adobe Photoshop and the blending mode of the fluorescence layer was set to “Color”. Note that no further image alignment was done in Photoshop. To enable visualization of both fluorescence and TEM images, the contrast levels of fluorescence images was linearly stretched when depicting CLEM overlays.

### Alignment accuracy estimation

Fiducial-based alignment accuracy was estimated using a leave-one-out cross validation (LOOCV) procedure^44^, as described in Kukulski *et al*.^6^. Briefly, up to 20 corresponding fiducial markers in the FLM and TEM images were selected manually, depending on the number of beads available. Next, successive alignments were performed using *n* − 1 (e.g., 19 out of 20) fiducial beads, and a rigid transformation was performed by the software, as described in Paul-Gilloteaux *et al*.^43^. The position of the omitted fiducial was then determined, in both FLM and TEM images, by determining the centre of gravity using a home-written Matlab (*Mathworks*) routine with sub-pixel accuracy. The difference between the predicted and true coordinate of each fiducial was determined for all beads, and the final alignment accuracy was estimated as the mean accuracy for all beads. To assess the alignment accuracy of FLM images of GFP to cryoFLM images of uranyl-stained sections, as well as cryoFLM images of uranyl-stained sections to the corresponding TEM images, we selected isolated and recognisable features (such as mitochondria and intracellular vesicles) that were visible in both image modalities. The image intensity along a line through the density was measured and the positions of full-width-half maximum (FWHM) on both sides of the structure were compared between cryoFLM and TEM. The difference between the FWHM location of the FLM and TEM profile on one side was calculated with single-pixel accuracy, and averaged with the difference of the other side to give the alignment accuracy. This was repeated for ten structures throughout each image, with various orientations of the profile line, to assess the accuracy across the whole image. The final alignment accuracy was calculated as the mean of the displacement of all ten positions. The overall alignment accuracy for GFP- or RFP-labelled structures to the TEM image was determined as described by Schellenberger *et al*.^12^, using the formula σ_overalll_ = √(σ_FP_^2^ + σ_uranyl_^2^), where σ_FP_ is the accuracy of the FP channel to the uranyl acetate image, and σ_uranyl_ is the accuracy of aligning the uranyl acetate image with the TEM image.

## Electronic supplementary material

SUPPLEMENTARY INFO
