# Supplementary material for: Inducing fluorescence of uranyl acetate as a dual-purpose contrast agent for correlative light-electron microscopy with nanometre precision
Source: Sci Rep. 2017 Sep 5;7:10442. doi: 10.1038/s41598-017-10905-x (PMC5585351; doi:10.1038/s41598-017-10905-x)
Supplement: Supplementary file 1 — SUPPLEMENTARY INFO [file 41598_2017_10905_MOESM1_ESM.pdf]

SUPPLEMENTARY INFORMATION

Inducing fluorescence of uranyl acetate as a dual-purpose contrast agent for correlative light-electron microscopy with nanometre precision

Maarten W. Tuijtel, Aat A. Mulder, Clara C. Posthuma, Barbara van der Hoeven, Abraham J. Koster, Montserrat Bárcena, Frank G. A. Faas* and Thomas H. Sharp*

*To whom correspondence should be addressed: F.G.A.Faas@lumc.nl, T.Sharp@lumc.nl

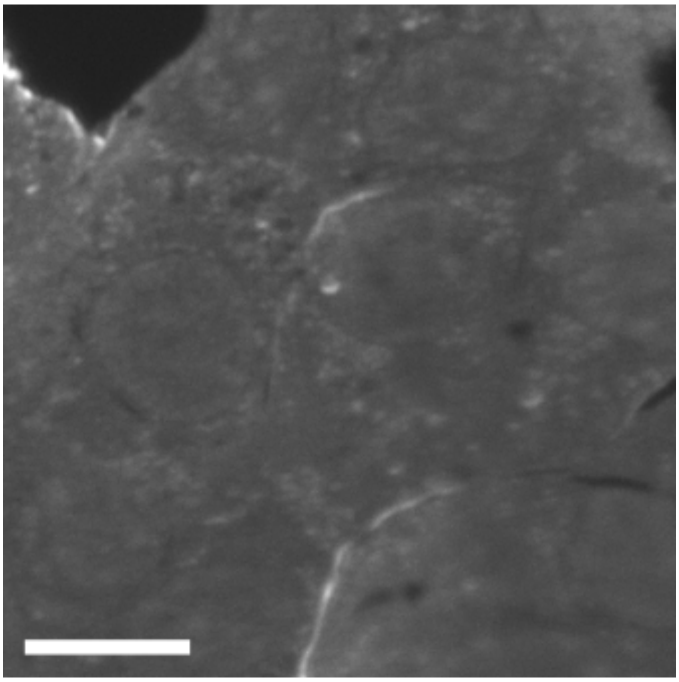

Supplementary Figure S1, related to Figure 1. CryoFLM image of a section prepared using the IRF sample protocol, without adding uranyl acetate to the freeze substitution mixture. Scale bar: 10 μm.

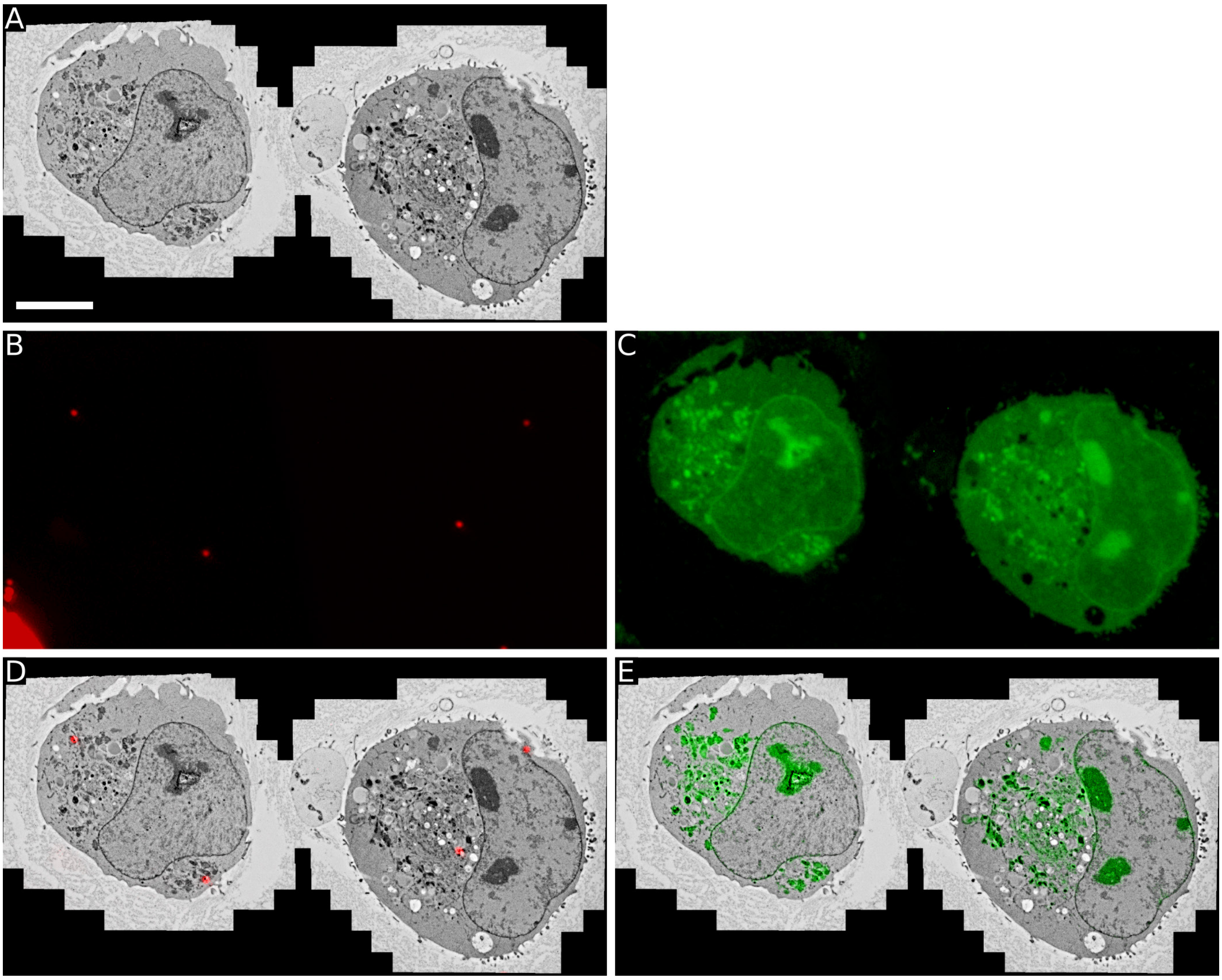

Supplementary Figure S2, related to Figure 3A. Uncropped version of **Fig. 3A**, with all four fiducial beads visible. **(A)** TEM image. **(B)** CryoFLM image of fiducial beads. **(C)** CryoFLM image of uranyl acetate stain. **(D & E)** Overlays of the fiducial beads (D) or the uranyl acetate stain (E) on the TEM image. Scale bar: 10 μm, applies to all panels.

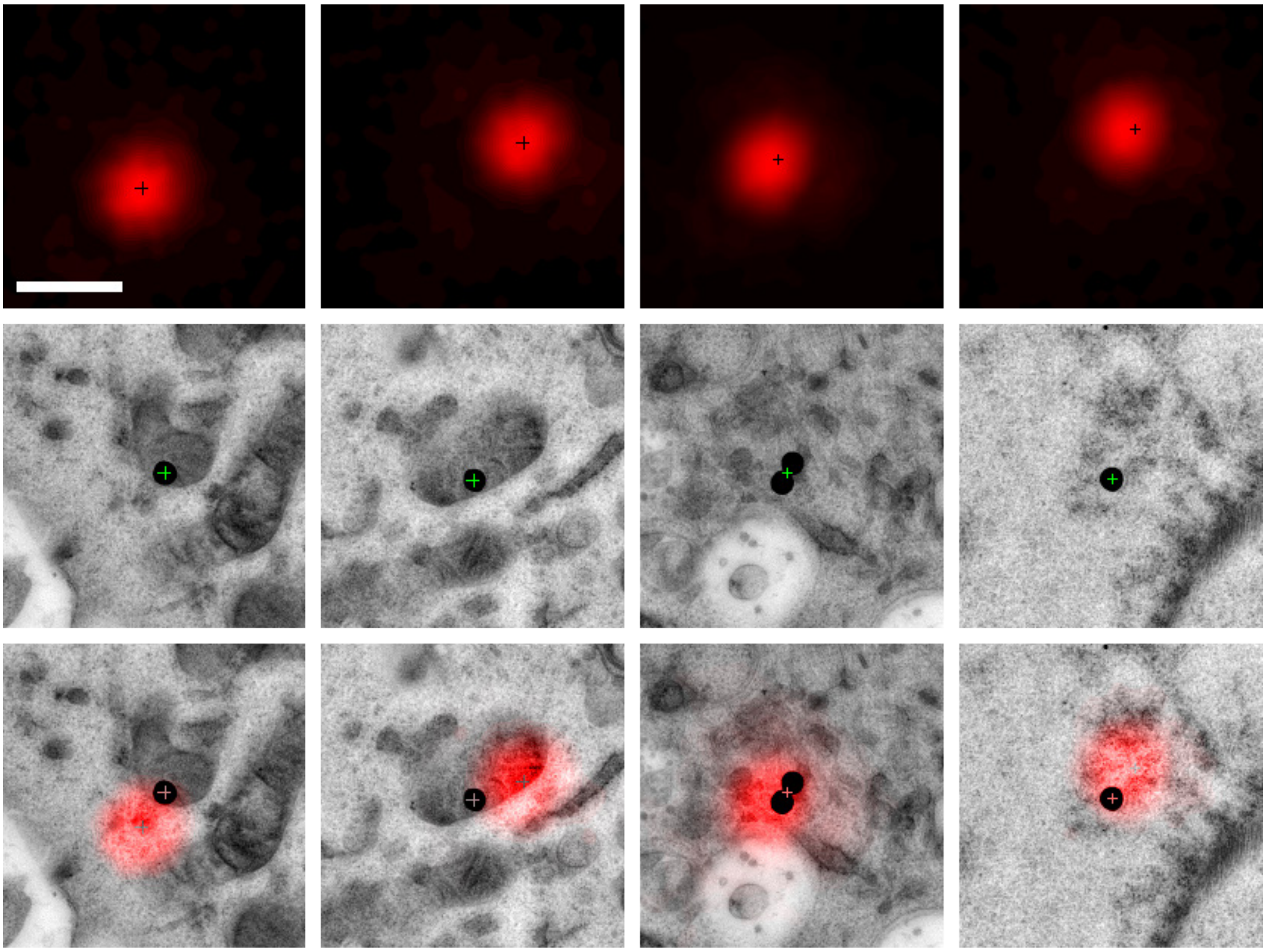

Supplementary Figure S3, related to Figure 3A. Measuring the accuracy of the fiducial-based alignment approach. All 4 fluorescent puncta (5 beads visible in the TEM) available are shown. Top row: CryoFLM image, with the calculated centre, based on the centre of gravity. Middle row: corresponding TEM image, with the calculated centre. Bottom row: overlay. Scale bar: 1 μm, applies to all panels.

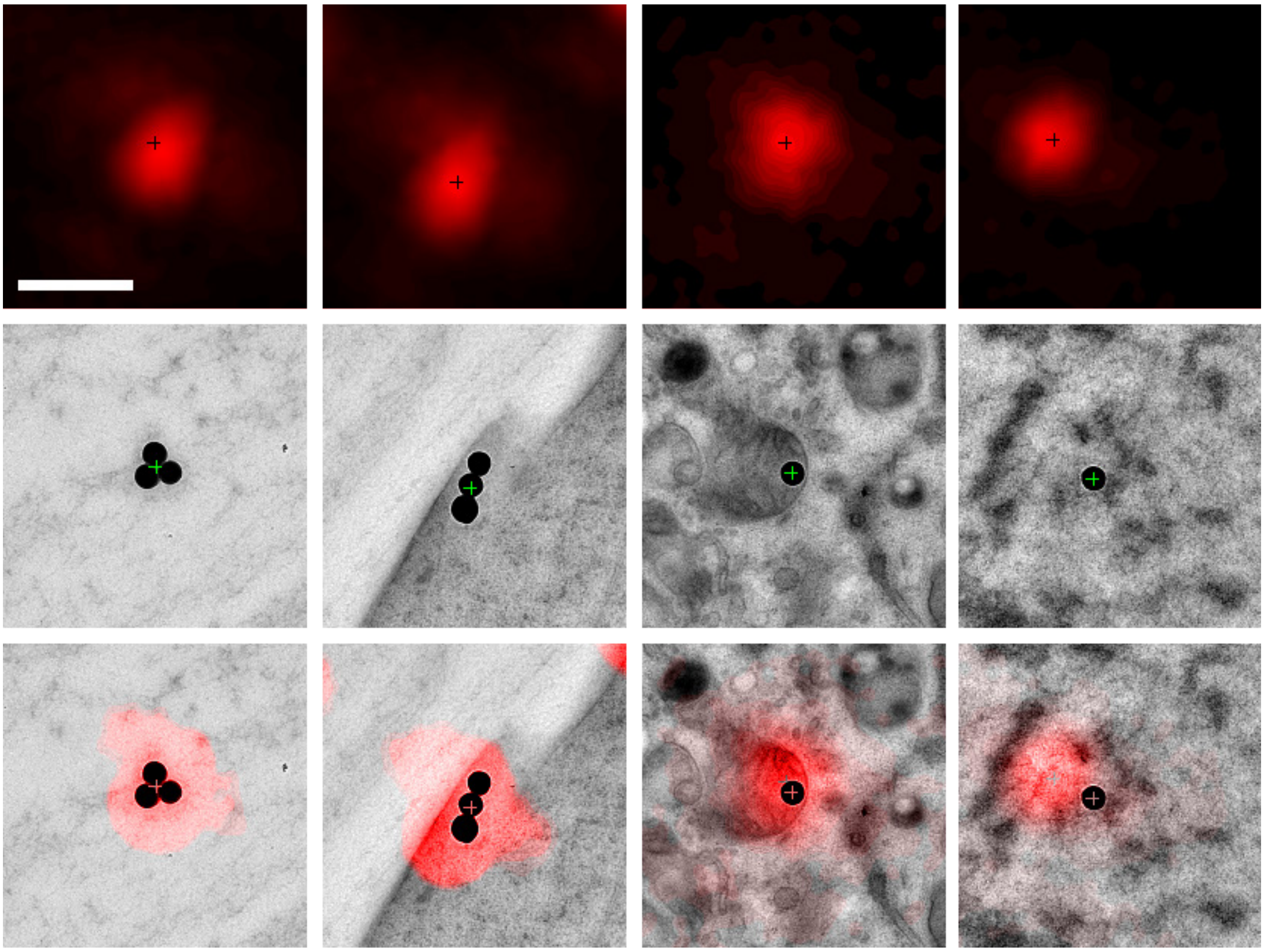

Supplementary Figure S4, related to Figure 3B. Measuring the accuracy of the fiducial-based alignment approach. A subset of 4 beads, out of a total of 20 picked, are shown. Top row: CryoFLM image, with the calculated centre, based on the centre of gravity. Middle row: corresponding TEM image, with the calculated centre. Bottom row: overlay. Scale bar: 1 μm, applies to all panels.

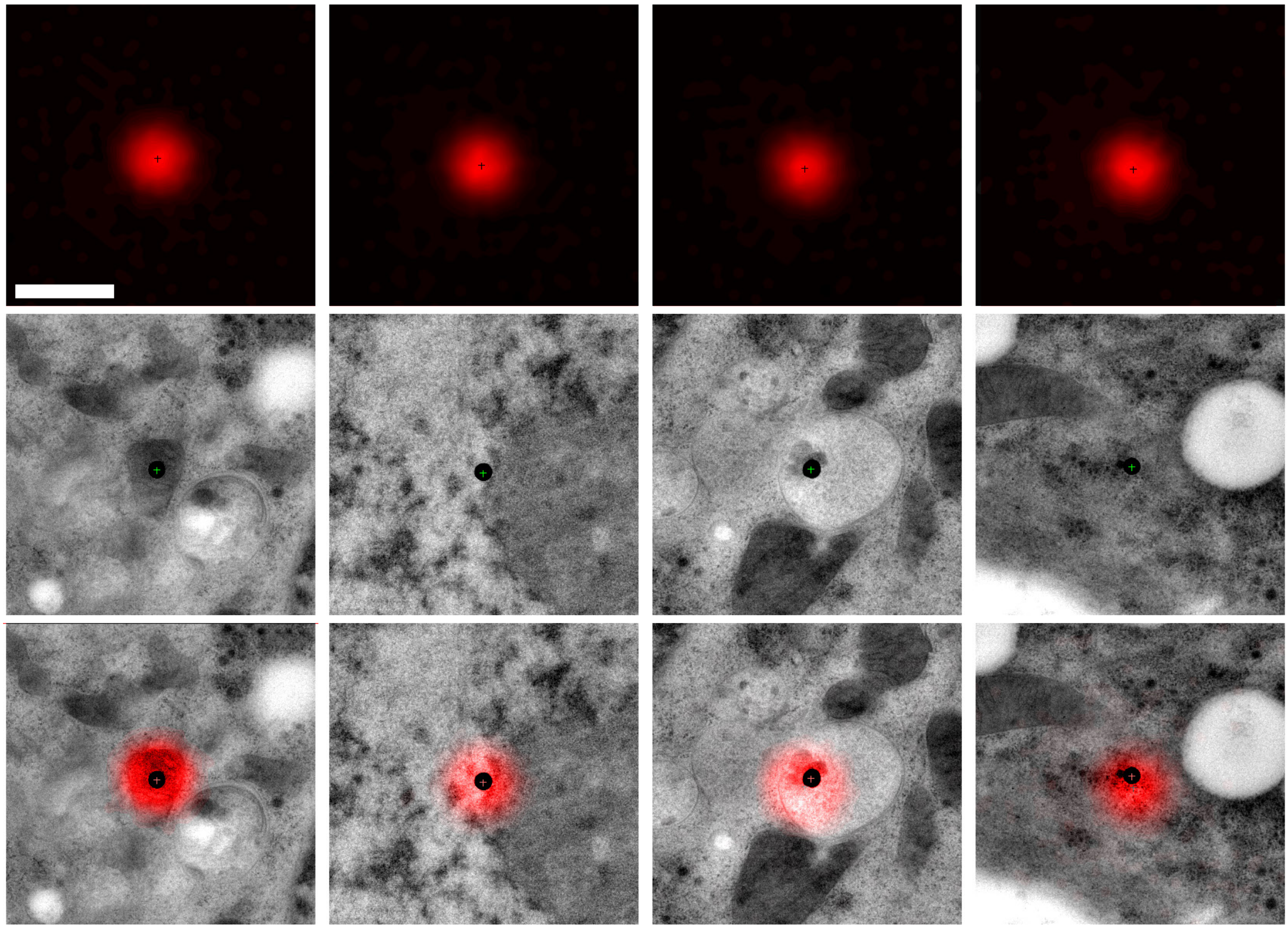

Supplementary Figure S5, related to Figure 3C. Measuring the accuracy of the fiducial-based alignment approach**.** Measuring accuracy of alignment based on fiducial beads on TEM images. A subset of 4 beads, out of a total of 20 picked are displayed. Top row: CryoFLM image, with the calculated centre, based on the centre of gravity. Middle row: corresponding TEM image, with the calculated centre. Bottom row: overlay. Scale bar: 1 µm, applies to all panels.

Supplementary Table S1. Estimating the accuracy of the fiducial-based alignment approach. The distance between the calculated centres of each fiducial measured in both cryoFLM and TEM images is displayed.

Distance between the calculated centres of fiducial beads measured in both cryoFLM and TEM images (nm)		
Figs. 3A & S3	Figs. 3B & S4	Figs. 3C & S5
371.1	352.1	63.4
500.2	103.4	46.3
105.0	144.9	56.3
355.9	69.2	40.0
	38.9	92.2
	42.3	51.0
	61.1	23.7
	127.4	34.7
	124.1	20.9
	42.3	35.0
	180.9	58.4
	115.2	110.9
	73.1	89.0
	127.4	120.9
	58.7	103.3
	189.4	74.2
	84.4	38.1
	61.6	68.3
	84.0	60.9
	163.3	85.6
Standard deviation (nm)		
165	73	29
Average displacement (nm)		
330	112	64

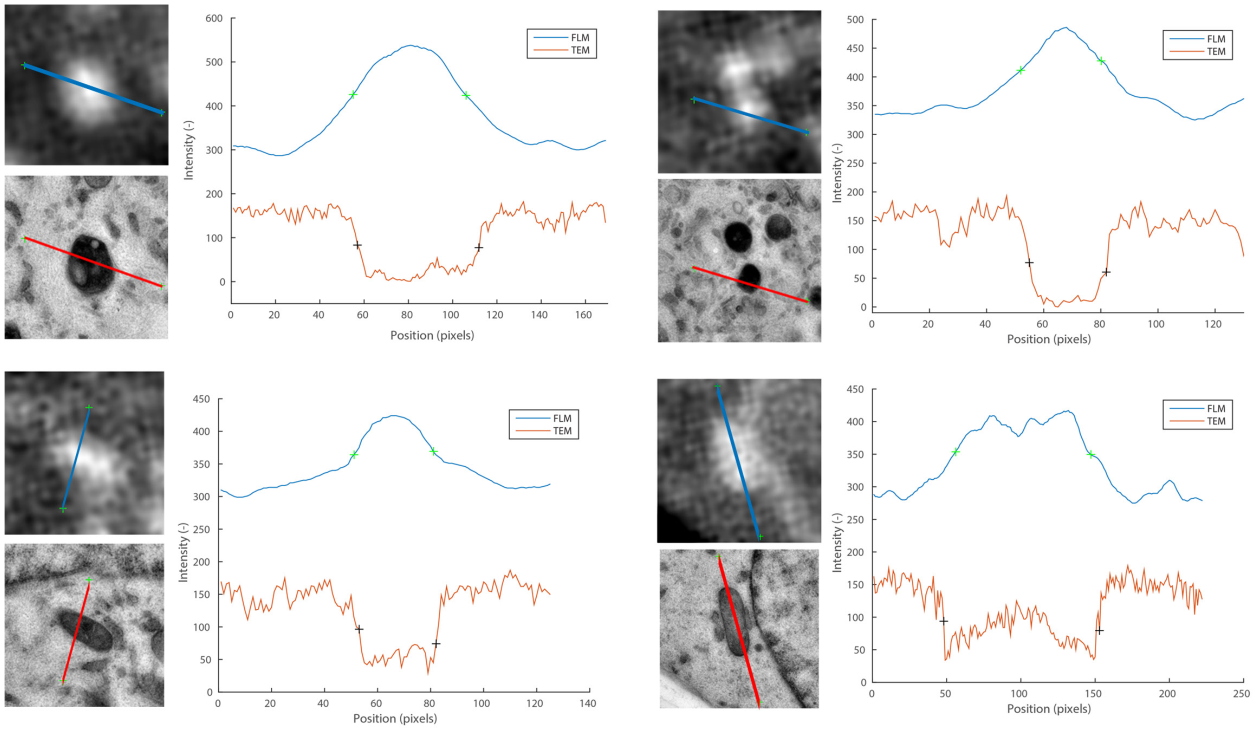

Supplementary Figure S6, related to Figure 3A. Estimating the accuracy of the uranyl acetate-based alignment approach. A subset of 4 of out of 10 chosen objects is depicted. For each panel: top left: CryoFLM image, bottom left: TEM image, with the profile depicted. Right: plot of the profile lines for FLM (blue) and TEM (red). The calculated FWHM for both modalities is indicated by crosses.

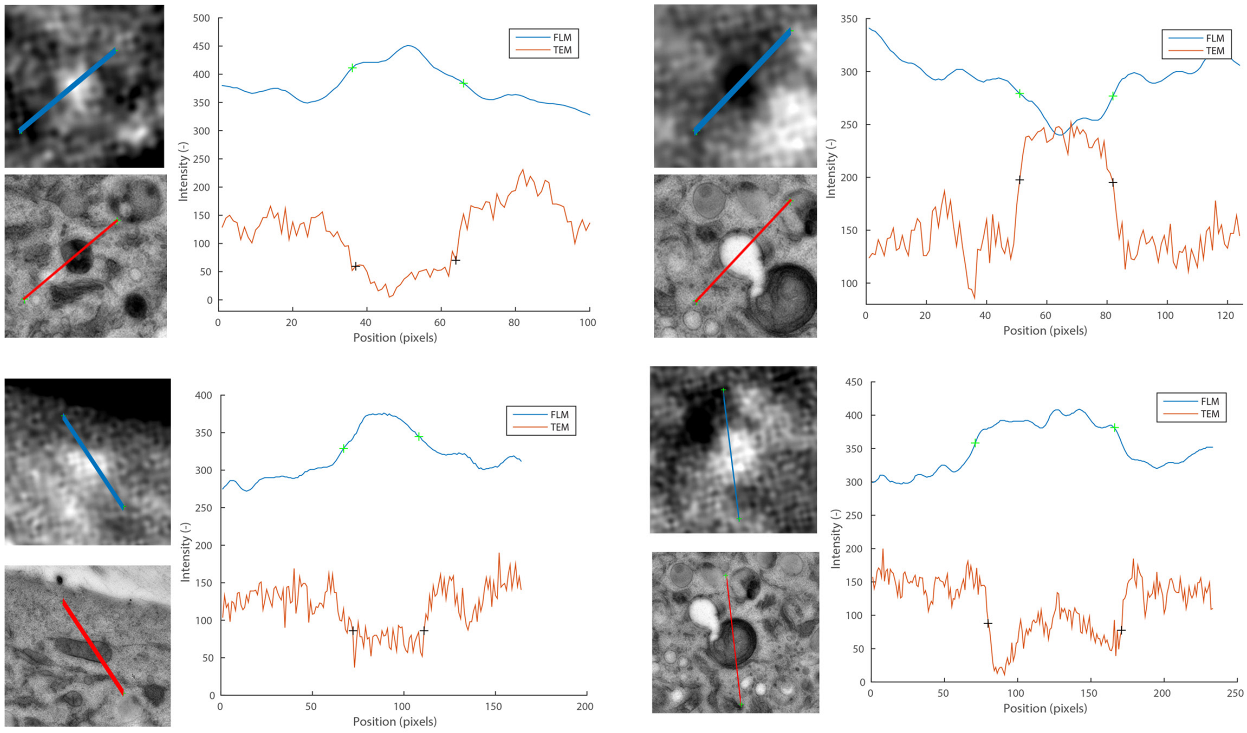

Supplementary Figure S7, related to Figure 3B. Estimating the accuracy of the uranyl acetate-based alignment approach. A subset of 4 of out of 10 chosen objects is depicted. For each panel: top left: CryoFLM image, bottom left: TEM image, with the profile depicted. Right: plot of the profile lines for FLM (blue) and TEM (red). The calculated FWHM for both modalities is indicated by crosses.

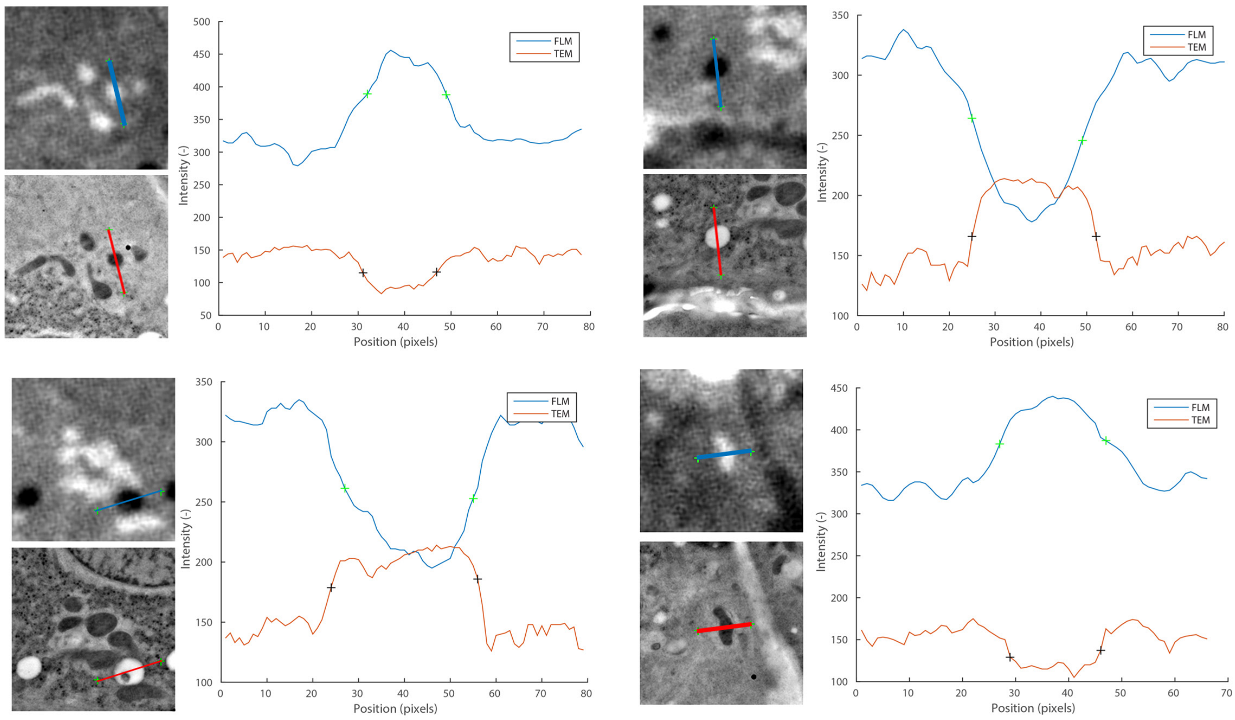

Supplementary Figure S8, related to Figure 3C. Estimating the accuracy of the uranyl acetate-based alignment approach. A subset of 4 of out of 10 chosen objects is depicted. For each panel: top left: CryoFLM image, bottom left: TEM image, with the profile depicted. Right: plot of the profile lines for FLM (blue) and TEM (red). The calculated FWHM for both modalities is indicated by crosses.

Supplementary Table S2. Estimating the accuracy of the uranyl acetate-based alignment approach. For each image pair, ten morphologically-discrete objects, such as mitochondria or intracellular vesicles, were selected. The difference between the FWHM as measured along the high-contrast edge of the selected objects for both imaging modalities are shown ($FWHM_{FLM}$ - $FWHM_{TEM}$), for both the left-hand side (lhs) and the right-hand side (rhs) of the selected objects.

Figs. 3A & S6			Figs. 3B & S7			Figs. 3C & S8		
lhs	rhs	|mean|	lhs	rhs	|mean|	lhs	rhs	|mean|
-2	-1	1.5	-5	-3	4.0	-6	-3	4.5
-6	-3	4.5	-1	-6	3.5	-11	-4	7.5
-2	-6	4.0	2	-3	0.5	-6	-1	3.5
-6	5	0.5	-9	-5	7.0	-9	3	3.0
0	2	0.5	-7	4	1.5	-4	-1	2.5
-3	-1	2.0	-2	5	1.5	-1	-5	3.0
8	-6	1.0	-1	2	0.5	-8	3	2.5
-3	-2	2.5	5	-9	2.0	3	-2	0.5
-3	0	1.5	-4	-1	2.5	2	5	3.5
-1	0	0.5	0	0	0.0	8	-3	2.5
Pixel size (nm)								
14.12			13.14			7.8		
Standard deviation (nm)								
20			28			15		
Average displacement (nm)								
26			30			26		

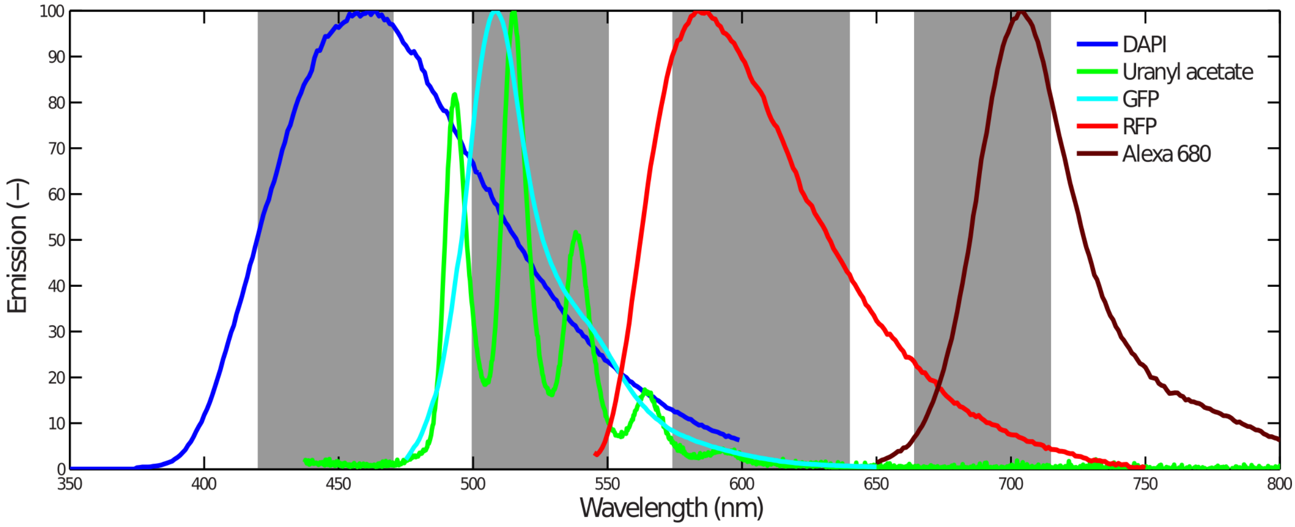

Supplementary Figure S9. The emission spectra of DAPI (blue), uranyl acetate (green), GFP (cyan), RFP (red) and Alexa Fluor 680 (brown), together with the common filter sets used (grey boxes) under cryogenic imaging conditions.

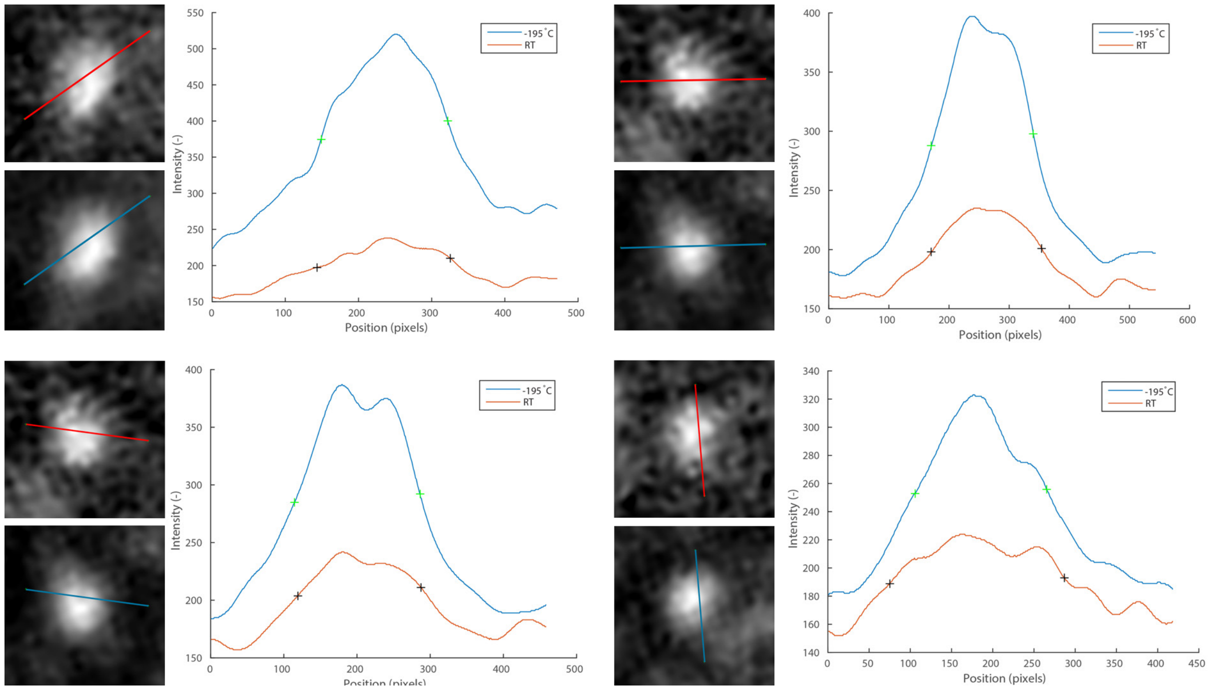

Supplementary Figure S10, related to Figure 4. Estimating the accuracy of the overlay of the uranyl acetate-based alignment approach. All 4 chosen objects are depicted. For each panel: top left: Room temperature (RT-) FLM image, bottom left: CryoFLM image, with the profile depicted. Right: plot of the profile lines for RT-FLM (red) and CryoFLM (blue). The calculated FWHM for both modalities is indicated by crosses.

Supplementary Table S3. Estimating the accuracy of alignment of the RT-FLM image of GFP to the CryoFLM image of uranyl acetate. Four mitochondria were selected. The difference between the FWHM as measured along the high-contrast edge of the selected objects for both imaging modalities are shown ($FWHM_{FLM}$ - $FWHM_{TEM}$), for both the left-hand side (lhs) and the right-hand side (rhs) of the selected objects.

Fig. 4		
lhs	rhs	|mean|
6	-4	1.0
0	-14	7.0
-12	15	1.5
-31	-22	4.5
Pixel size (nm)		
3.4		
Standard deviation (nm)		
10		
Average displacement (nm)		
12		

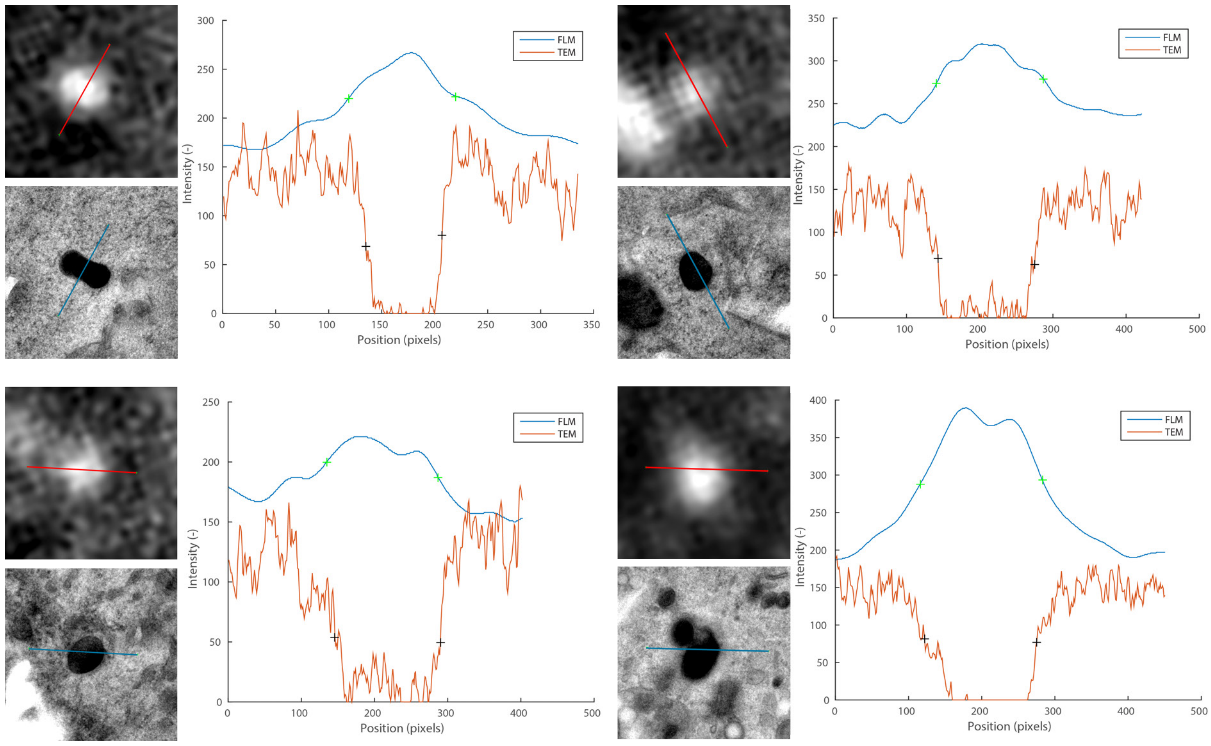

Supplementary Figure S11, related to Figure 4. Estimating the accuracy of the uranyl acetate-based alignment approach. A subset of 4 of out of 10 chosen objects is depicted. For each panel: top left: CryoFLM image, bottom left: TEM image, with the profile depicted. Right: plot of the profile lines for FLM (blue) and TEM (red). The calculated FWHM for both modalities is indicated by crosses.

Supplementary Table S4. Estimating the accuracy of the uranyl acetate-based alignment approach. For each image pair, ten morphologically-discrete objects, such as mitochondria or intracellular vesicles, were selected. The difference between the FWHM as measured along the high-contrast edge of the selected objects for both imaging modalities are shown ($FWHM_{FLM}$ - $FWHM_{TEM}$), for both the left-hand side (lhs) and the right-hand side (rhs) of the selected objects.

Fig. 4			Fig. 5			Fig. 6		
lhs	rhs	|mean|	lhs	rhs	|mean|	lhs	rhs	|mean|
-16	13	1.5	-7	8	0.5	0	-2	1.0
-2	12	5.0	1	10	5.5	-3	0	1.5
-35	44	4.5	-4	0	2.0	-7	-4	5.5
-11	-3	7.0	-5	-12	8.5	0	2	1.0
-6	8	1.0	-1	5	2.0	-2	6	2.0
-5	20	7.5	11	5	8.0	5	4	4.5
-6	-35	20.5	2	9	5.5	2	12	7.0
-27	20	3.5	4	-5	0.5	-1	-8	4.5
-9	11	1.0	-2	6	2.0	6	3	4.5
-37	19	9.0	7	2	4.5	-10	0	5.0
Pixel size (nm)								
3.4			9.8			9.8		
Standard deviation (nm)								
20			29			21		
Average displacement (nm)								
21			38			36		

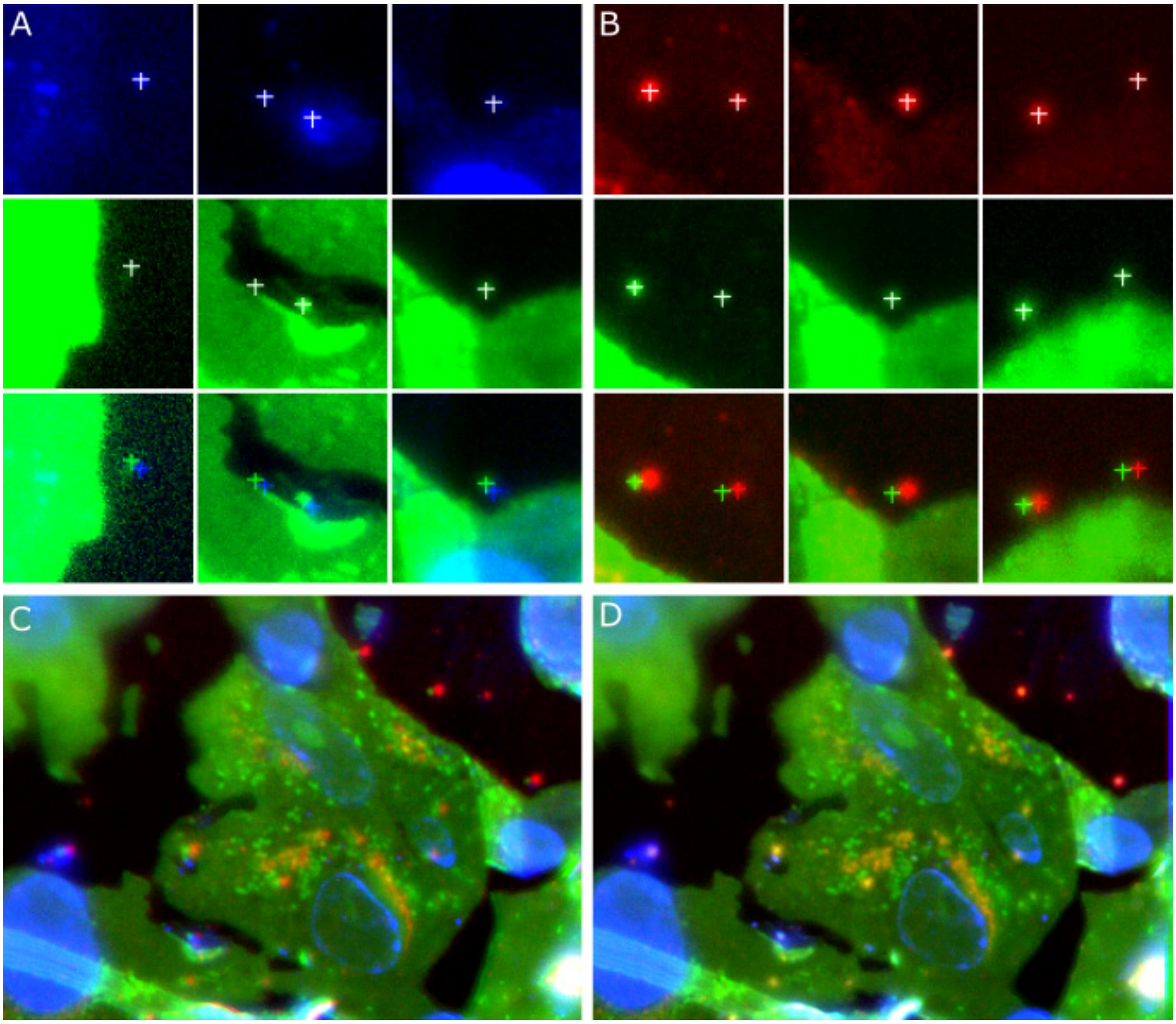

Supplementary Figure S12, related to Figure 5. CryoFLM images showing the colour alignments necessary for the DAPI, uranyl acetate and RFP channels. **(A)** Alignment of DAPI (top) and uranyl acetate (middle) images, with overlay (bottom). **(B)** Alignment of RFP (top) and uranyl acetate (middle) images, with overlay (bottom). **(C)** Non-aligned image stack. **(D)** Aligned image stack.

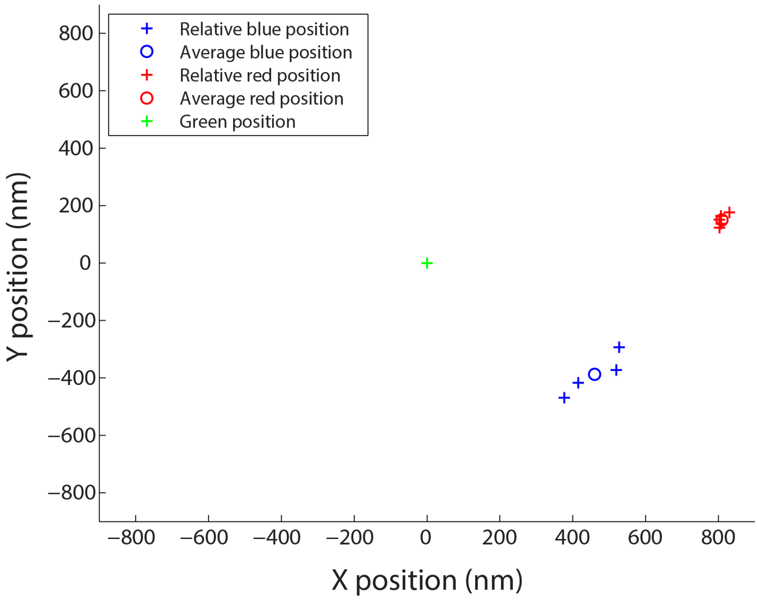

Supplementary Figure S13, related to Figure 5 and Figure S12. Estimation of accuracy of alignment of different colour channels, as depicted in **Figure S12**. The relative position of each structure shown in **Figure S12A & B** with respect to the same structure in the uranyl acetate channel (blue and red crosses, respectively). The average position used for image alignment is also shown (blue and red circles). Alignment accuracy was estimated by the standard deviation and yielded 106 nm and 24 nm (for the DAPI and RFP channel, respectively.

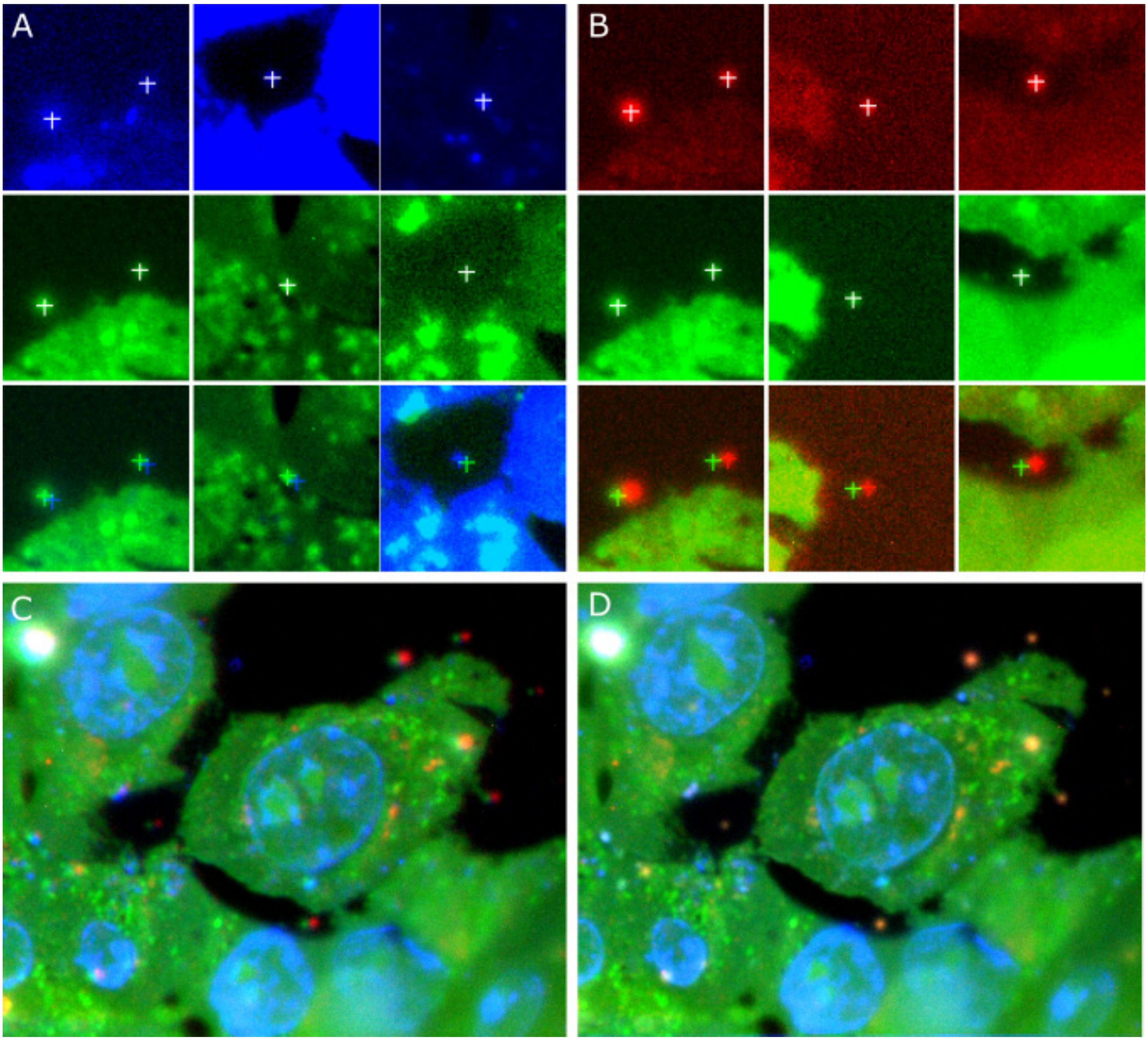

Supplementary Figure S14, related to Figure 6. CryoFLM images showing the colour alignments necessary for the DAPI, uranyl acetate and RFP channels. **(A)** Alignment of DAPI (top) and uranyl acetate (middle) images, with overlay (bottom). **(B)** Alignment of RFP (top) and uranyl acetate (middle) images, with overlay (bottom). **(C)** Non-aligned image stack. **(D)** Aligned image stack.

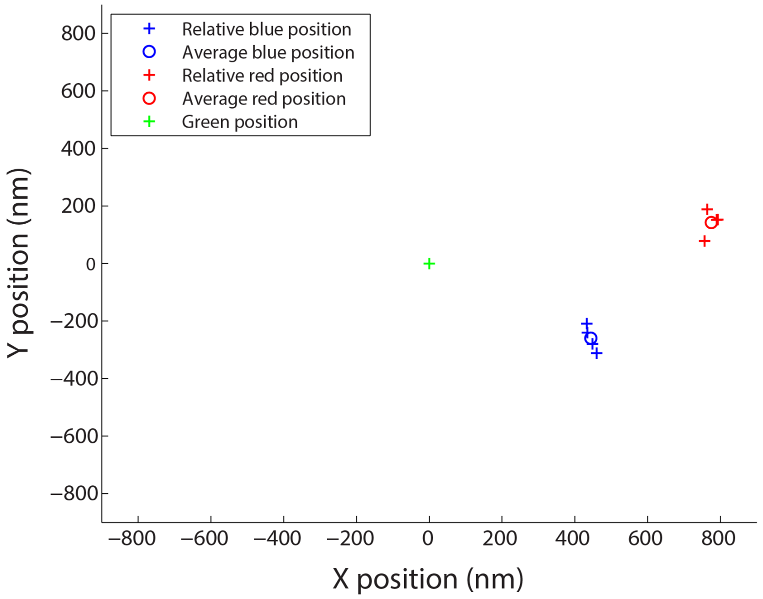

Supplementary Figure S15, related to Figure 6 and Figure S14. Estimation of accuracy of alignment of different colour channels, as depicted in **Figure S14**. The relative position of each structure shown in **Figure S14A & B** with respect to the same structure in the uranyl acetate channel (blue and red crosses). The average position used for image alignment is also shown (blue and red circles). Alignment accuracy was estimated by the standard deviation and yielded 47 nm and 50 for the DAPI and RFP channel, respectively.

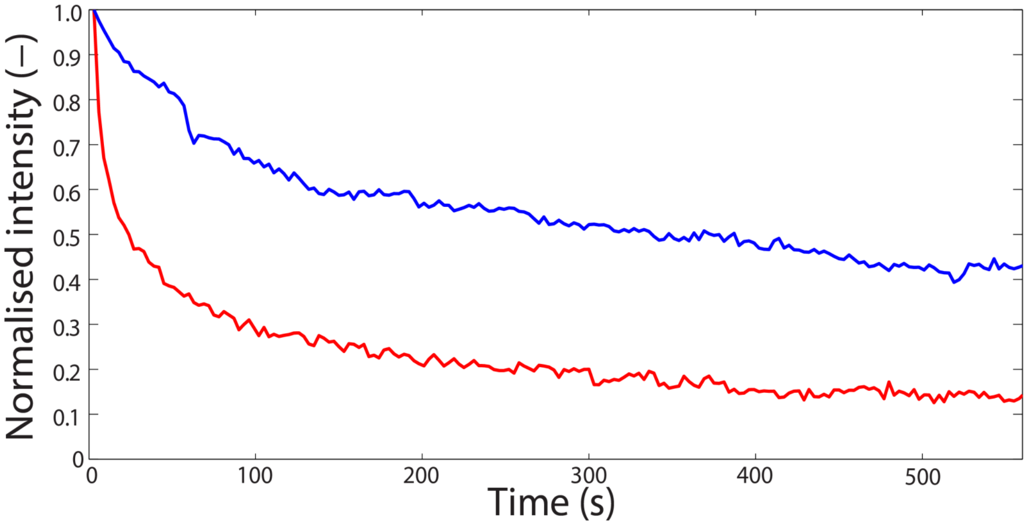

Supplementary Figure S16. The fluorescence-bleaching rate of RFP is significantly reduced at -195°C (blue line) compared to 21°C (red line).
